# SPLICE-q: a Python tool for genome-wide quantification of splicing efficiency

**DOI:** 10.1186/s12859-021-04282-6

**Published:** 2021-07-15

**Authors:** Verônica R. de Melo Costa, Julianus Pfeuffer, Annita Louloupi, Ulf A. V. Ørom, Rosario M. Piro

**Affiliations:** 1 Institute of Computer Science and Institute of Bioinformatics, Freie Universität Berlin, Berlin, Germany; 2 Department of Computational Molecular Biology, Max Planck Institute for Molecular Genetics, Berlin, Germany; 3 Department of Computer Science, Eberhard Karls Universität Tübingen, Tübingen, Germany; 4 Institute for Bioinformatics and Medical Informatics Tübingen, Eberhard Karls Universität Tübingen, Tübingen, Germany; 5 Max Delbrück Center for Molecular Medicine, Berlin Institute for Medical Systems Biology, Berlin, Germany; 6 Department of Molecular Biology and Genetics, Aarhus University, Aarhus, Denmark; 7 Dipartimento di Elettronica, Informazione e Bioingegneria, Politecnico di Milano, Milan, Italy

**Keywords:** Splicing efficiency, RNA-seq, Co-transcriptional splicing, Splicing dynamics

## Abstract

**Background:**

Introns are generally removed from primary transcripts to form mature RNA molecules in a post-transcriptional process called splicing. An efficient splicing of primary transcripts is an essential step in gene expression and its misregulation is related to numerous human diseases. Thus, to better understand the dynamics of this process and the perturbations that might be caused by aberrant transcript processing it is important to quantify splicing efficiency.

**Results:**

Here, we introduce SPLICE-q, a fast and user-friendly Python tool for genome-wide **SPLIC**ing **E**fficiency **q**uantification. It supports studies focusing on the implications of splicing efficiency in transcript processing dynamics. SPLICE-q uses aligned reads from strand-specific RNA-seq to quantify splicing efficiency for each intron individually and allows the user to select different levels of restrictiveness concerning the introns’ overlap with other genomic elements such as exons of other genes. We applied SPLICE-q to globally assess the dynamics of intron excision in yeast and human nascent RNA-seq. We also show its application using total RNA-seq from a patient-matched prostate cancer sample.

**Conclusions:**

Our analyses illustrate that SPLICE-q is suitable to detect a progressive increase of splicing efficiency throughout a time course of nascent RNA-seq and it might be useful when it comes to understanding cancer progression beyond mere gene expression levels. SPLICE-q is available at: https://github.com/vrmelo/SPLICE-q

**Supplementary Information:**

The online version contains supplementary material available at 10.1186/s12859-021-04282-6.

## Background

Eukaryotic genes are mostly composed of a series of exons intercalated by sequences with no coding potential called introns. These sequences are generally removed from primary transcripts by a post-transcriptional process called splicing to form mature RNA molecules. This highly regulated process consists basically of a series of hydrolysis and ligation reactions led by the spliceosome [1]. The exon–intron boundaries, i.e., the splice junctions, together with the branch point, a short sequence located 18–40 nucleotides upstream of the intron's 3' splice junction [2] and the polypyrimidine tract [3], are recognized by the spliceosome. These events promote the correct folding necessary for the intron’s excision and are followed by the correct pairing of the exon-exon boundaries. In metazoans, further sequences are required for recruiting different trans-acting regulatory factors. These will act as spliceosome regulators as well as splice site choice modulators and are particularly important for efficient transcript processing [4].

Splicing is dynamic and occurs mostly during or immediately after the transcription of a complete intron. Co-transcriptional splicing was first suggested in *D. melanogaster* chorion genes using electron microscopy to observe the assembly of spliceosomes at the splice junctions in nascent transcripts [5]. More recently, genome-wide studies in different cell lines and organisms using nascent RNA showed introns being spliced shortly after their transcription is finished: in *S. cerevisiae*, data revealed polymerase pausing within the terminal exon, permitting enough time for splicing to happen before release of the mature RNA [6]; and nascent RNA also indicated that most introns in *D. melanogaster* are co-transcriptionally spliced [7], as well as in mouse [8] and many human cells and tissues [9–11].

Splicing is an essential step in gene expression and its misregulation is related to numerous human diseases [12–15]. Up to 15% of mutations that cause genetic disease have been suggested to affect pre-mRNA splicing [16]. Thus, to better understand the dynamics of splicing and the perturbations that might be caused by aberrant transcript processing, it is important to quantify splicing efficiency. The efficiency of splicing is commonly quantified by means of RT-qPCR with primers that span exon-exon and exon–intron boundaries [17]. Yet, this methodology can only investigate a limited number of genes. By contrast, transcriptomics technologies, such as RNA-Seq, allow these analyses from a genome-wide point of view. One interesting approach to globally determine splicing efficiencies is to assess nascent transcripts within short intervals after the transcription has started. Experimentally, this can be achieved through metabolic labeling or purification of chromatin-associated nascent RNAs.

For intron-containing transcripts, splicing efficiency can be determined with different frameworks that use read counts on intronic and exonic regions. Short-read RNA-Seq is currently the main approach using either nascent or total RNA. Conceptually, splicing efficiency can be observed either from an intron-centric point of view—to investigate whether an intron has been spliced out—or from an exon-centric point of view—to investigate whether an exon has been correctly spliced within the context of its transcript, or to what degree an exon is included in the transcript molecules generated from a gene.

Khodor et al. [7] used an intron-centric method to estimate the unspliced fraction of introns in *D. melanogaster* by taking the ratio of the read coverage of the last 25 bp of an intron and the first 25 bp of the following exon. In this way, introns where the RNA polymerase has not yet reached the acceptor splice site are not included but the metric is not guaranteed to take values between 0 and 1 and does hence not constitute an efficiency metric in the strict sense. Tilgner et al. [10] used deep-sequencing of human subcellular fractions and developed an exon-centric “completed splicing index” (coSI) which takes reads spanning the 5’ and the 3’ splice junctions of an exon and computes the fraction of reads indicating completed splicing, i.e., which span from exon to exon, to study co-transcriptional splicing. By explicitly considering also reads which span from the upstream exon directly to the downstream exon, this approach includes exon skipping events, but coSI values for the first and last exon of a transcript cannot be determined. More recently, Převorovský et al. [18] presented a workflow for genome-wide determination of intron-centric splicing efficiency in yeast. The efficiencies are quantified for the 5’ and 3’ splice junctions separately as the number of “*transreads*” (split reads spanning from exon to exon) divided by the number of reads covering the first or last base of the intron, respectively. Although the authors call their metric “splicing efficiency”, it is not limited to a range from 0 to 1 and it is not clear how cases without intronic reads (divisions by zero) are handled. Other drawbacks of this workflow are that it consists of numerous open-source tools and custom shell and R scripts and that it was explicitly developed for yeast.

In contrast to these approaches, IRFinder [19] was designed to detect intron retention from mRNA-seq data by measuring a so-called intron retention ratio (*IR ratio*) based on the average read coverage in a given intron and the number of split reads at the corresponding intron–exon junctions. While the approach is similar to the one we present here, it has the opposite goal: to determine the degree to which introns are retained rather than spliced out. Lastly, iREAD [20] has the same goal as IRFinder, but instead of providing a quantitative measure for intron retention levels, it makes binary judgments (considering introns as either retained or not retained) based on a set of features, including intron expression levels.

Although the above-mentioned frameworks for calculating splicing efficiency from RNA-seq data exist, there is more to add to their respective limitations. The bioinformatics steps involved might be challenging—including difficulties in running workflows that require long running times and the installation of numerous tools—especially for experimental biologists. Thus, here we introduce SPLICE-q, a user-friendly open-source Python tool for genome-wide **SPLIC**ing **E**fficiency **q**uantification from RNA-seq data. SPLICE-q quantifies splicing efficiency for each intron individually and allows the user to select different levels of restrictiveness concerning an intron’s overlap with other genomic elements. We show the usefulness of SPLICE-q by applying it to time-series nascent and steady-state RNA-seq data from human and yeast.

## Implementation

### SPLICE-q workflow and parameters

**Fig. 1:**
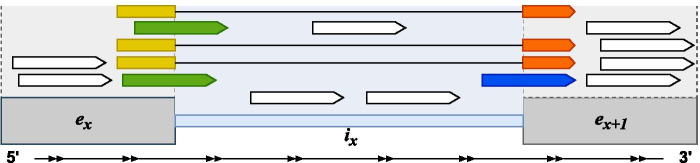
Read assignment scheme for splicing efficiency (*SE*) and inverse intron expression ratio (*IER*). Illustration of the reads used by SPLICE-q to quantify *SE* and *IER*. In yellow, split reads at the 5’ splice junction; in orange, split reads at the 3’ splice junction; in green, unsplit reads at the 5’ splice junction; in dark blue, unsplit reads at the 3’ splice junction. In gray and blue, the areas covering the exons and introns, respectively. In white, reads not overlapping splice junctions

SPLICE-q is a tool, implemented in Python 3, for quantification of individual intron splicing efficiencies from strand-specific RNA-seq data. SPLICE-q's main quantification method uses splicing reads—both split and unsplit—spanning the splice junctions of a given intron (Fig. 1). Split reads are junction reads spanning from one exon to another, thus indicating processed transcripts from which the individual intron has already been excised. Intuitively, unsplit reads are those spanning the intron–exon boundaries (covering both sides of the splice junction), hence, indicating transcripts from which the intron has not yet been spliced out. As an alternative measure for splicing efficiency, SPLICE-q computes an inverse intron expression ratio, which compares the introns’ expression levels with those of their flanking exons.

SPLICE-q is also sensitive to the overlap of genomic elements. In other words, SPLICE-q takes into consideration when a genome shows overlapping features that can cause issues with a correct assignment of reads to specific introns or exons. For example, for intron–exon boundaries overlapping exons of other genes, seemingly unsplit reads might instead stem from exonic regions of the overlapping genes. This is problematic due to the RNA-seq methodology’s limitation that makes it difficult to confidently determine without ambiguity to which genomic element, exon or intron, these reads should be attributed [21].

**Fig. 2:**
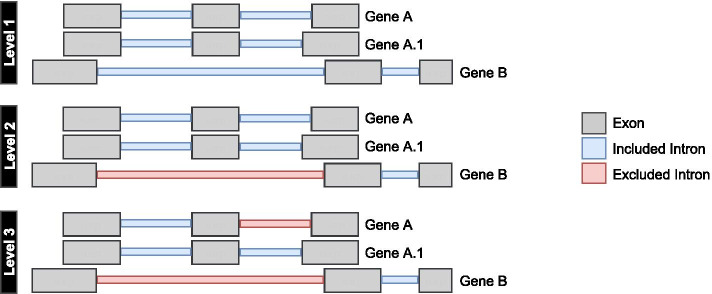
SPLICE-q’s levels of restrictiveness. (Level 1) keep all introns in the genome regardless of overlaps with other genomic elements; (Level 2) select only introns whose splice junctions do not overlap any exon of a different gene; (Level 3) select only introns that do not overlap with any exon of the same or a different gene. A and A.1 are isoforms of the same gene (A) and B represents a different gene

Therefore, SPLICE-q allows the user to select different levels of restrictiveness for strand-specific filtering, including (1) Level 1: keep all introns in the genome regardless of overlaps with other genomic elements; (2) Level 2: select only introns whose splice junctions do not overlap any exon of a different gene; (3) Level 3: select only introns that do not overlap with any exon of the same or a different gene (Fig. 2). These levels to not affect computed splicing efficiency measures, but instead determine for which set of introns the splicing efficiencies are to be quantified. Other filters, including the minimum read coverage at splice junctions, can also be set up according to users’ necessities (Additional file 1: Table S1).

The two necessary input files are:

1. A Binary Alignment Map (BAM) file with RNA-seq reads aligned to the reference genome.
2. A genome annotation file provided by sources like GENCODE [22] or Ensembl [23] in Gene Transfer Format (GTF) containing information on exons and the genes and transcripts they are associated with.

**Fig. 3:**
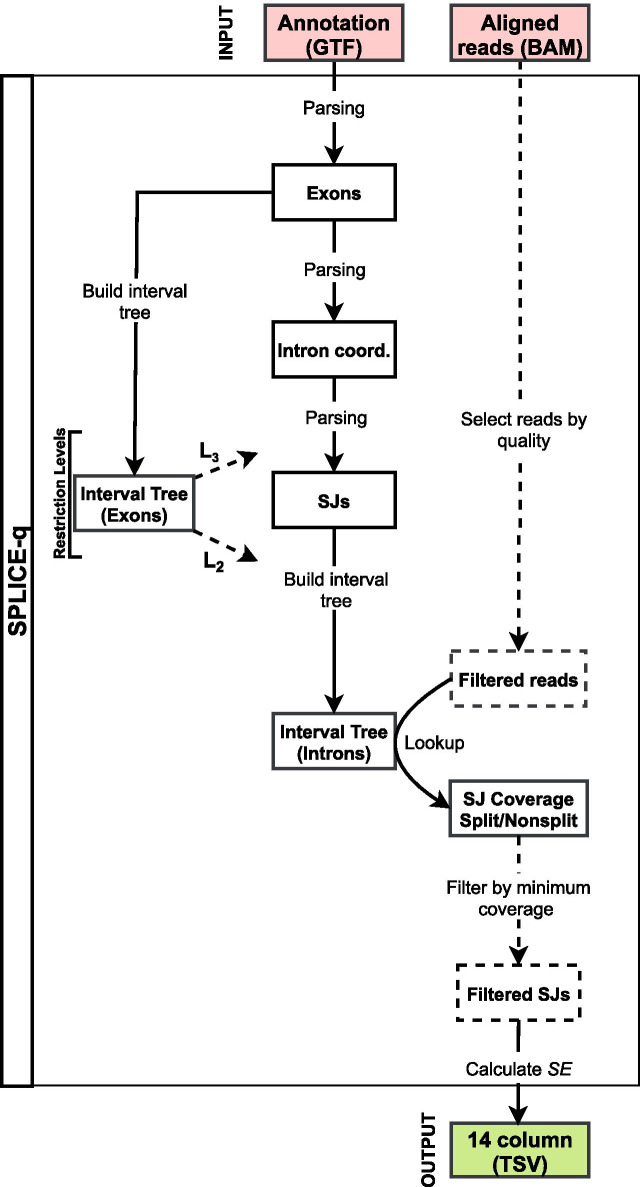
SPLICE-q’s default workflow. Dashed lines indicate steps which depend on parameter settings. Solid lines represent the mandatory steps of the workflow. Boxes illustrate data types: input (red), intermediate data items (white) and output (green). SJ = splice junction; TSV = tab-separated values. Levels of restrictiveness: L_2_ (Level 2) and L_3_ (Level 3)

SPLICE-q’s internal default workflow comprises of the following major steps (Fig. 3):

1. Parsing of genomic features from the GTF file;
2. Locating and annotating introns and splice junctions from the GTF file’s exon coordinates;
3. Filtering of introns according to the level of restrictiveness based on the overlap of genomic elements;
4. Selection of split and unsplit reads at the splice junctions according to the reads’ concise idiosyncratic gapped alignment report (CIGAR), and subsequent coverage calculation for each splice junction.
5. Computation of splicing efficiencies.

SPLICE-q parses the exon-centric GTF file and infers the corresponding intron coordinates, partially adapting related functions of GTFtools [24]. For Level 3 filtering, when the user chooses to include the inverse intron expression ratio, the workflow includes two additional steps (Additional file 1: Fig. S1):

6. Computation of median per-base coverages of introns and their flanking exons
7. Computation of the inverse intron expression ratios.

### Quantifying splicing efficiency and inverse intron expression ratio

*Splicing efficiency (SE):* SPLICE-q uses split and unsplit junction reads to quantify *SE* for each intron individually. It determines the RNA-seq reads mapping to both splice junctions of each given intron *i*, distinguishes split (*S*) and unsplit (*N*) reads for the 5’ and 3’ splice junctions and estimates a splicing efficiency score (*SE*_*i*_) as a function of the corresponding read counts as follows:

1 $$SE_{i} = \frac{{\mathop \sum \nolimits_{{j \in \left\{ {5^{\prime},3^{\prime}} \right\}}} S_{i}^{j} }}{{\mathop \sum \nolimits_{{j \in \left\{ {5^{\prime},3^{\prime}} \right\}}} \left( {S_{i}^{j} + N_{i}^{j} } \right)}}\quad 0 \le SE_{i} \le 1$$

Since the purpose of *SE*_*i*_ is to measure the fraction of spliced transcripts with respect to a given intron *i*, the 5'- and 3'-split reads (${S}_{i}^{5{^{\prime}}}$ and ${S}_{i}^{3{^{\prime}}}$) explicitly include all reads which span from the respective exon junction to any other exon, thus including alternatively spliced reads, not only those reads which span directly from the flanking upstream to the flanking downstream exon. In other words, spliced reads are also counted if they indicate that more than intron *i* was spliced out. An *SE* of 0 indicates that the intron has not been spliced out in any of the transcripts from which the junction reads originate, which may be due to late splicing in case of nascent RNA-seq or intron retention in case of steady-state RNA-seq. An *SE* of 1 means completed splicing on all transcripts. Therefore, *SE* values ranging between 0 and 1 approximate the fraction of molecules which have already been spliced. This quantification method makes it possible to compare spliced and unspliced intron rates directly.

*Inverse intron expression ratio (IER)* as an alternative measure for splicing efficiency when using Level 3 filtering, SPLICE-q also provides the inverse of the ratio of intron expression to exon expression, where *I*_*x*_ is the median per-base read coverage of the *x*-th intron of a given transcript and *E*_*x*_ and *E*_*x*+*1*_ represent the corresponding median coverages of the flanking exons:

2 $$IER = 1 - min\left( {1,\frac{{I_{x} }}{{0.5 \cdot \left( {E_{x} + E_{x + 1} } \right)}}} \right)\quad 0 \le IER \le 1$$

Here, the focus lies specifically on the per-base median coverage of all reads mapping to the involved genomic elements (exonic and intronic reads) rather than just the splice junctions (Fig. 1). As explained above, a high *SE* indicates that an intron was spliced out of a large fraction of transcripts. This scenario should display high read coverage in the exons and low coverage or none in the intron. In other words, peaks of mapped reads are observed in the surrounding exons when compared to the intron itself. On the contrary, introns with a low *SE* should have read coverage profiles more similar to the surrounding exons.

## Results and discussion

### Fast and user-friendly quantification of splicing efficiency

Due to its simplicity and efficient data structure for working with genomic intervals, SPLICE-q’s run time with default parameters for approximately 100 million input reads mapped to the human genome is 18 min using a MacBook Pro with a dual-core Intel Core i5 processor and 8 GB of RAM. By increasing the number of processes to 4 or 8 (see the command line parameter *NProcesses* described in Additional file 1: Table S1), which is not an issue considering nowadays’ number of processor cores of most laptops and desktops, the running time on an AMD Opteron 6282 SE with 516 GB of memory is less than 2 min (Additional file 1: Fig. S2a). Memory usage is low, being approximately that of the GTF file size (1.4 GB for the human genome; Additional file 1: Fig. S2b). SPLICE-q’s approach provides major advantages over previous workflows which may require the installation of numerous tools and suffer from long running times (see Additional file 2).

### Splicing kinetics in human and yeast

**Fig. 4:**
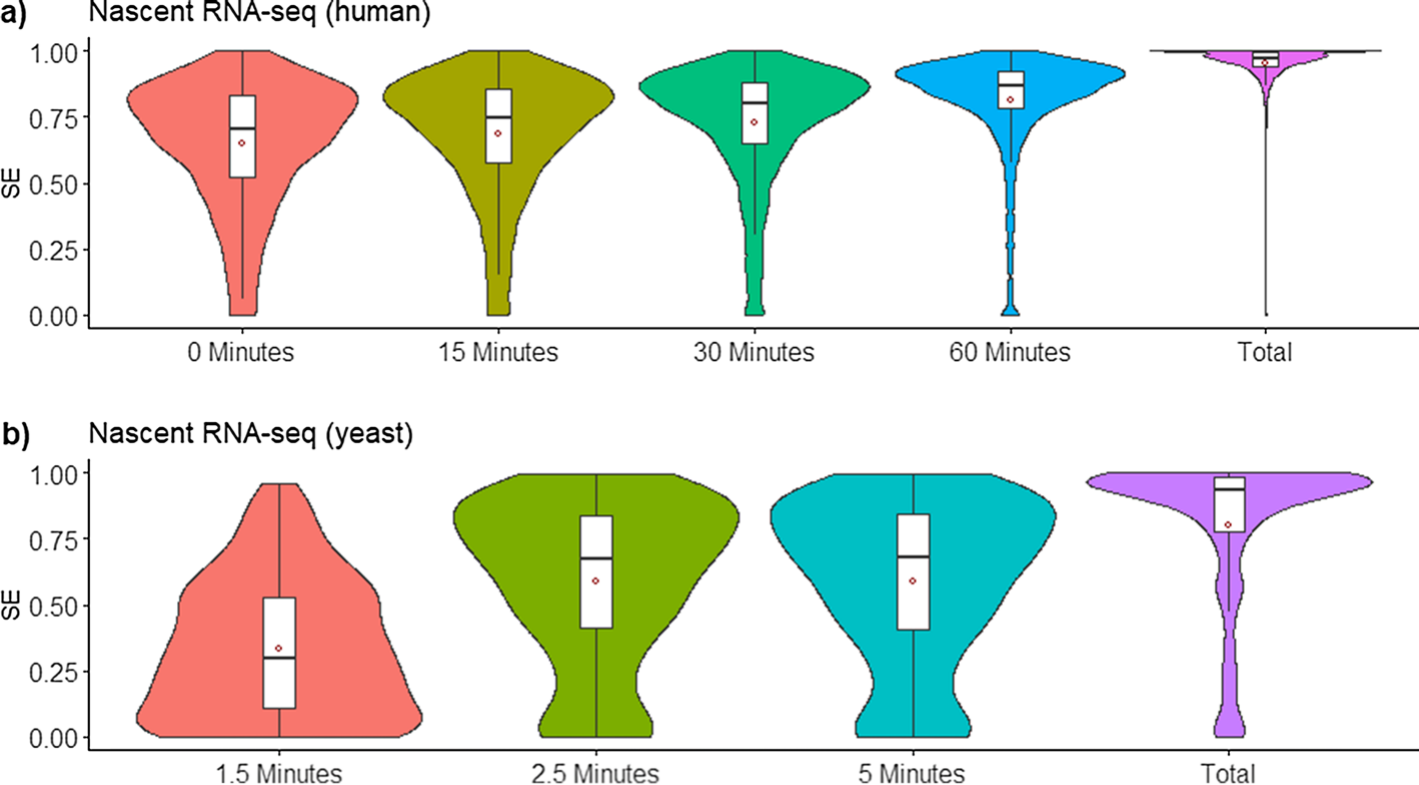
Splicing kinetics using different datasets. **a** Time-series nascent and steady-state (total) RNA-seq of labeled HEK293 cells; **b** Time-series nascent and steady-state RNA-seq of *S. cerevisiae*

We applied SPLICE-q to globally assess the kinetics of intron excision. The goal here is to show the tool’s applicability using different data. For this purpose, we performed three different analyses using data from two species and different methodologies (Additional file 1: Materials and Methods). The first time-series sequencing dataset consists of BrU-labeled HEK293 cells with 15 min pulse labeling of nascent RNA and subsequent sequencing of labeled RNA after 0, 15, 30, and 60 min (pulse-chase) [25]. On average we obtained ~ 200 million reads per sample, ~ 85% of which were uniquely mapped. The nascent RNA samples were compared to an unlabeled steady-state control of the same cell line [26]. SPLICE-q was applied with default parameters: filtering level 3, a minimum coverage of 10 uniquely mapped reads at each splice junction, and a minimum intron length of 30 nucleotides [27]. Only introns passing the filters in all samples after running SPLICE-q were kept, totalizing 13,178 introns. As expected, SPLICE-q detects a progressive increase of *SE* throughout the time course (Fig. 4a). Interestingly, at 0 and 15 min, *SE* scores are already high with a median of 0.71 and 0.75, respectively.

The increase of splicing efficiency over the time course can also be observed when *IER* values are computed for the same data (Additional file 1: Fig. S3). For all time points, the median *IER* scores are higher than the median *SE* scores (0.82, 0.84, 0.86, 0.92 and 1 for 0, 15, 30, 60 min and steady-state control, respectively). While there are notable differences between *SE* and *IER* scores for individual introns, overall, the two measures of splicing efficiency are strongly correlated (Additional file 1: Fig. S4-5).

The results for both *SE* and *IER* agree with previous studies showing that splicing is predominantly co-transcriptional in humans and for the most part happens immediately after the transcription of an intron is completed, when the RNA polymerase has proceeded only a few bases into the downstream exon [5, 6, 9–11]. However, the results also illustrate that even 60 min after the pulse-labeling of newly synthesized RNA, there is a significantly larger fraction of introns which have not yet been excised from the transcripts than in the steady-state control.

We chose a second dataset [28] which would allow us to quantify splicing efficiency of nascent RNA within a finer time scale. These sequencing experiments were performed with 4-thiouracil labeled RNA (4tU-seq) from *Saccharomyces cerevisiae*. Nascent RNA was labeled for an extremely short time (1.5, 2.5 and 5 min) and then sequenced (Fig. 4b). Unlabeled control samples were also generated. After alignment of the raw data, we obtained an average of over 50 million uniquely mapped reads per sample and 246 introns shared between all samples after running SPLICE-q with the above-mentioned default parameters and filtering level 2. The *SE* at 1.5 min has a median of 0.29 while, strikingly, there is an increase of 131% in just 1 min, with a median *SE* of 0.67 at 2.5 min. This value does not alter in the next time point and the unlabeled control sample shows a median *SE* of 0.93. While, due to the differences in experimental protocols, the results obtained for the second dataset are not directly comparable to those observed for HEK293 cells, this brief analysis suggests how essential it is to perform short labeling in *S. cerevisiae* in order to assess its splicing kinetics since some transcripts approximate steady-state levels in a time as short as 2.5 min (Fig. 4b).

Lastly, we show how SPLICE-q can also be applied to quantify intron retention in steady-state RNA-seq data. For this purpose, we used data coming from a prostate cancer sample along with its matched normal tissue (patient 15 of ref. [29]). Since for each of the tissues two replicates were available, we computed splicing efficiencies for each replicate and then averaged the results for the tumor tissue and the normal tissue.

**Fig. 5:**
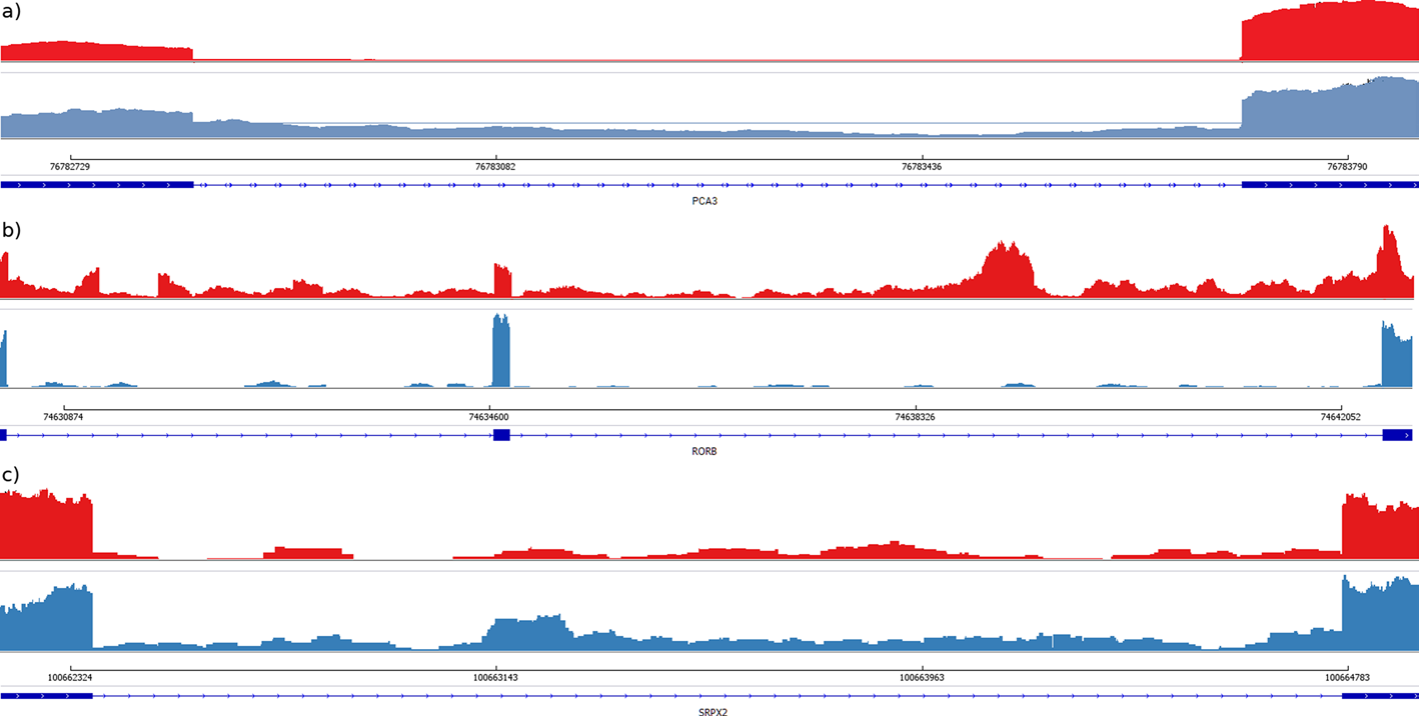
Read coverage of selected introns in the prostate cancer and the normal control sample. IGV views of representative cases of introns from different genes comparing prostate cancer versus normal samples. **a** Intron located at chr9:76,782,833–76,783,704 of PCA3; **b** Introns located at chr9:74,630,368–74,634,630 and chr9:74,634,773–74,642,413 of RORB; and **c** Intron located at chrX:100,662,368–100,664,773 of SRPX2. Tumor and normal samples are represented in red and blue, respectively. The figure shows only one of the two sample replicates. For both replicates, see Additional file 1: Fig. S8

Prostate cancer is one of the most common cancer types in men [30]. SPLICE-q detected relatively high splicing efficiencies—median *SE* of 0.96 in both the tumor and the normal sample—in the 66,389 introns shared across the sample pair after running the tool with default parameters. This is expected when the tool is applied to steady-state RNA-seq data. The *SE* and *IER* scores for individual introns are strongly correlated, both among each other (Additional file 1: Fig. S6-7, Table S2) and across sample replicates (Additional file 1: Fig. S9). Although this overview suggests that there is no alteration in average splicing efficiency levels between normal and tumor tissue, a closer look showed interesting changes for individual introns. One intriguing example is *Prostate cancer associated 3* (PCA3), a long noncoding RNA highly expressed in prostate cancer and widely known as a prostate cancer-specific biomarker of high specificity [31]. It has been found to be involved in the proliferation and survival of prostate cancer cells by multiple mechanisms, including the modulation of androgen receptor signaling, the inhibition of the tumor suppressor PRUNE2, and possibly by acting as a competing endogenous RNA (ceRNA) for *High mobility group box 1* (HMGB1) via sponging of *miR-218-5p* [31–33]. Interestingly, PCA3’s second intron located at chr9:76,782,833–76,783,704 has an *SE* of 0.57 in normal tissue and a much higher *SE* of 0.90 in the tumor (Fig. 5a), suggesting that PCA3 might not only be overexpressed but also more efficiently spliced.

Variation in splicing efficiency can be also observed among protein coding genes. The *retinoic acid-related orphan receptor β (*RORβ, encoded by the gene RORB) was recently reported to inhibit tumorigenesis in colorectal cancer in vivo. When RORβ was overexpressed, tumorigenic capacity of the cells was significantly reduced, suggesting that this protein acts as a tumor suppressor in colorectal cancer [34]. Intriguingly, we found two of the RORB introns—located at chr9:74,630,368–74,634,630 and chr9:74,634,773–74,642,413—to have reduced splicing efficiencies in the prostate cancer sample (*SEs* of 0.99 and 0.98 in the normal control and 0.63 and 0.60 in the tumor, respectively) (Fig. 5b). Contrasting, *Sushi repeat-containing protein X‐linked 2*, or simply SRPX2, shows the opposite splicing efficiency profile with an intron at the coordinates chrX:100,662,368–100,664,773 being less efficiently spliced in the control sample (*SE* of 0.59) than in the tumor (*SE* of 0.90, Fig. 5c). Previous studies showed SRPX2 to play an important role in cancer development and progression. In colorectal cancer, the overexpression of SRPX2 may promote invasiveness of tumor cells [35], and in prostate cancer, a knockdown of SRPX2 affected the proliferation, migration and invasion of cancer cells by partially suppressing the PI3K/Akt/mTOR signaling pathway [36]. PI3K/Akt/mTOR regulates cell proliferation and survival in different cancer types and is usually activated in advanced prostate cancer [37, 38]. Furthermore, the suppression of this signaling pathway was reported to reduce cell motility and invasion in prostate cancer [39].

While these examples obtained from a single prostate tumor provide no biological insight into prostate cancer in general, they illustrate that gene regulation may go beyond the mere expression levels, with a gain or loss of splicing efficiency potentially acting as a superposed mechanism that may be beneficial to tumor development.

## Comparison with existing methods

We compared SPLICE-q to other methods for the analysis of splicing efficiency or intron retention, namely iREAD [20], IRFinder [19], and a workflow implemented by Převorovský et al. [18]. As outlined above, and described in more detail in Additional file 2, the purpose of iREAD is to determine whether an intron can be considered as retained or not. Also, IRFinder detects intron retention but provides a quantitative measure, called *IR ratio*, which describes the degree of intron retention. This is similar to the intention of our *SE* and *IER* scores, but follows the inverse logic. To our best knowledge, apart from SPLICE-q only Převorovský et al.’s workflow was designed to calculate “splicing efficiencies”. We compared SPLICE-q to these methods both with respect to the computational performance and the obtained measurements.

For performance comparison, we used either the HEK293 nascent RNA dataset or the prostate cancer sample and its matched control. Both SPLICE-q's run time and memory footprint were superior to those of the other tested methods, especially when compared to iREAD and Převorovský et al.’s workflow (see Additional file 2).

To compare the obtained intron retention and splicing efficiency scores, we first examined whether the other approaches provide results which are correlated to SPLICE-q’s *SE* and *IER*. While the results obtained from IRFinder are conceptually different from (or even opposite to) the notion of splicing efficiency, as expected they prove to be correlated when considering the inverse of the IR ratio (1-*IRratio*), as shown in Figures S2 and S3 in Additional file 2. Although iREAD provides only binary decisions regarding intron retention—it provides no quantitative degree that would be directly comparable to *SE* or *IER* scores—the fraction of detected intron retention events decreases with increasing *SE* or *IER*, as anticipated. However, the overall fraction of introns identified by iREAD remains rather low (Fig. S4 in Additional file 2) due to its restrictive default thresholds for detection of intron retention. Neither IRFinder nor iREAD were able to detect the events we described for the prostate tumor in Fig. 5 (see Additional file 2: Section 6).

Also the scores computed by Převorovský et al.’s workflow are correlated with splicing efficiencies (at least on a logarithmic scale, see Fig. S1 in Additional file 2) but they are not limited to a range from 0 to 1 and do hence not directly measure the degree to which an intron is spliced out, which makes them less interpretable. Moreover, depending on the use case (e.g., the species of interest), running the workflow will likely require manual modification of the source code (see Additional file 2 for some necessary modifications).

Lastly, we compared the three tools with respect to their capability to identify simulated events of increasing intron retention levels, that is, decreasing splicing efficiency. While iREAD turned out to be too restrictive when using default parameters, SPLICE-q and IRFinder provide comparable results, although SPLICE-q is slightly better when it comes to recognizing borderline cases at lower coverage. For further details regarding these comparisons and justification for our conclusions, please see Additional file 2.

Altogether, SPLICE-q clearly outperforms iREAD and Převorovský et al.’s workflow and shows slight improvements over IRFinder. Moreover, SPLICE-q is easier to install and use than the other tools and provides two alternative measurements for evaluating splicing efficiency as well as a larger set of filtering options.

## Conclusions

Here we introduced SPLICE-q, an efficient and user-friendly tool for splicing efficiency quantification. SPLICE-q enables the quantification of splicing through two different methods (*SE* and *IER*) and is sensitive to the overlap of genomic elements. We demonstrated SPLICE-q’s usefulness showing three use cases, including two different species and experimental methodologies. Our analyses illustrate that SPLICE-q is suitable to detect a progressive increase of splicing efficiency throughout a time course of strand-specific nascent RNA-seq data. Likewise, SPLICE-q can be applied to strand-specific steady-state RNA-seq data and might be useful when it comes to understanding cancer progression beyond mere gene expression levels, for example, by identifying differential splicing efficiency between tumor samples and their respective normal controls.

## Supplementary Information

**Additional file 1**: Supplementary figures, tables and materials and methods. The file provides supplementary figures and tables regarding SPLICE-q’s workflow, parameters, run time and memory usage, and additional results, including direct comparisons of SE and IER scores. It also describes the experimental and computational protocols used for BrU-chase RNA sequencing.

**Additional file 2**: Usage examples and performance evaluation for SPLICE-q. The file describes SPLICE-q’s basic usage and obtained output. It further provides information on SPLICE-q’s performance as compared to other tools and workflows, including a simulation of retention levels or reduced splicing efficiency.

## Data Availability

The datasets generated and/or analyzed during the current study can be downloaded from the NCBI Gene Expression Omnibus repository with accession numbers GSE92565, GSE83561, GSE84722, GSE70378 and GSE133626. **Availability and requirements**: Project name: SPLICE-q, Project homepage: https://github.com/vrmelo/SPLICE-q. Operating system (s): Linux, macOS, and Windows 10 Subsystem for Linux. Programming language: Python 3, Other requirements: Python 3.x., including packages PySam and InterLap. License:GNU GPL-2, Any restrictions to use by non-academics: None.

## Abbreviations

4tU: 4-Thiouracil
Akt: Protein kinase B
BAM: Binary alignment map
BrU: Bromouridine
ceRNA: Competing endogenous RNA
CIGAR: Concise idiosyncratic gapped alignment report
coSI: Completed splicing index
GTF: Gene transfer format
HMGB1: High mobility group box 1
IER: Inverse intron expression ratio
mTOR: Mechanistic/mammalian target of rapamycin
PCA3: Prostate cancer associated 3
PI3K: Phosphoinositide 3-kinase
RORβ/RORB: Retinoic acid-related orphan receptor β
RT-qPCR: Quantitative reverse transcription polymerase chain reaction
SE: Splicing efficiency
SJ: Splice junction
SPLICE-q: SPLICing efficiency quantification
SRPX2: Sushi repeat-containing protein X‐linked 2
TSV: Tab-separated values
Bp: Base pair
HEK293: Human embryonic kidney 293
IGV: Integrative genomics viewer
Pre-mRNA: Precursor messenger RNA
PRUNE2: Protein prune homolog 2
RNA: Ribonucleic acid
RNA-seq: RNA sequencing
