# Supplementary material for: SPLICE-q: a Python tool for genome-wide quantification of splicing efficiency
Source: BMC Bioinformatics. 2021 Jul 15;22:368. doi: 10.1186/s12859-021-04282-6 (PMC8281633; doi:10.1186/s12859-021-04282-6)
Supplement: Supplementary file 1 — Additional file 1: Supplementary figures, tables and materials and methods. The file provides supplementary figures and tables regarding SPLICE-q’s workflow, parameters, run time and memory usage, and additional results, including direct comparisons of SE and IER scores. It also describes the experimental and computational protocols used for BrU-chase RNA sequencing. [file 12859_2021_4282_MOESM1_ESM.pdf]

Additional File 1

Supplementary Tables, Figures and Materials and Methods

SPLICE-q: a Python tool for genome-wide quantification of splicing efficiency

Verônica R Melo Costa[1,2,*], Julianus Pfeuffer[1,3,4], Annita Louloupi[5], Ulf A V Ørom[6], Rosario M Piro[7,*]

[1] Institute of Computer Science and Institute of Bioinformatics, Freie Universität Berlin, Berlin, Germany

[2] Department of Computational Molecular Biology, Max Planck Institute for Molecular Genetics, Berlin, Germany

[3] Department of Computer Science, Eberhard Karls Universität Tübingen, Tübingen, Germany

[4] Institute for Bioinformatics and Medical Informatics Tübingen, Eberhard Karls Universität Tübingen, Tübingen, Germany

[5] Berlin Institute for Medical Systems Biology, Max Delbrück Center for Molecular Medicine, Berlin, Germany

[6] Department of Molecular Biology and Genetics, Aarhus University, Aarhus, Denmark

[7] Dipartimento di Elettronica, Informazione e Bioingegneria, Politecnico di Milano, Milan, Italy

* Correspondence

veronica.melocosta@gmail.com

rosariomichael.piro@polimi.it

Table S1: Summary table of parameters.

Parameter	Description
MinCoverage	Minimum number of reads spanning each splice junction (Default = 10).
MinReadQuality	Mapping quality. By default, only uniquely mapped reads are included (Default = 10).
MinIntronLength	Minimum intron length. Default value is optimal for analysis using human RNA-seq data (Default = 30)
ChromsList	List of chromosome names (Default: chr1-720, I-XVI, 2L, 2R, 3L, 3R, Z, W.)
FilterLevel	(1) keep all introns in the genome regardless of overlaps with other genomic elements.<br>(2) select only introns whose splice junctions do not overlap any exon in different genes<br>(3) select only introns that do not overlap with any exon of the same or different gene (Default).
IERatio	Running mode that additionally outputs the Inverse Intron Expression Ratio (IER). Requires *FilterLevel* 3.
NProcesses	Multiple concurrent processes are used to minimize running times and the number of processes can be adjusted by the user through this parameter.

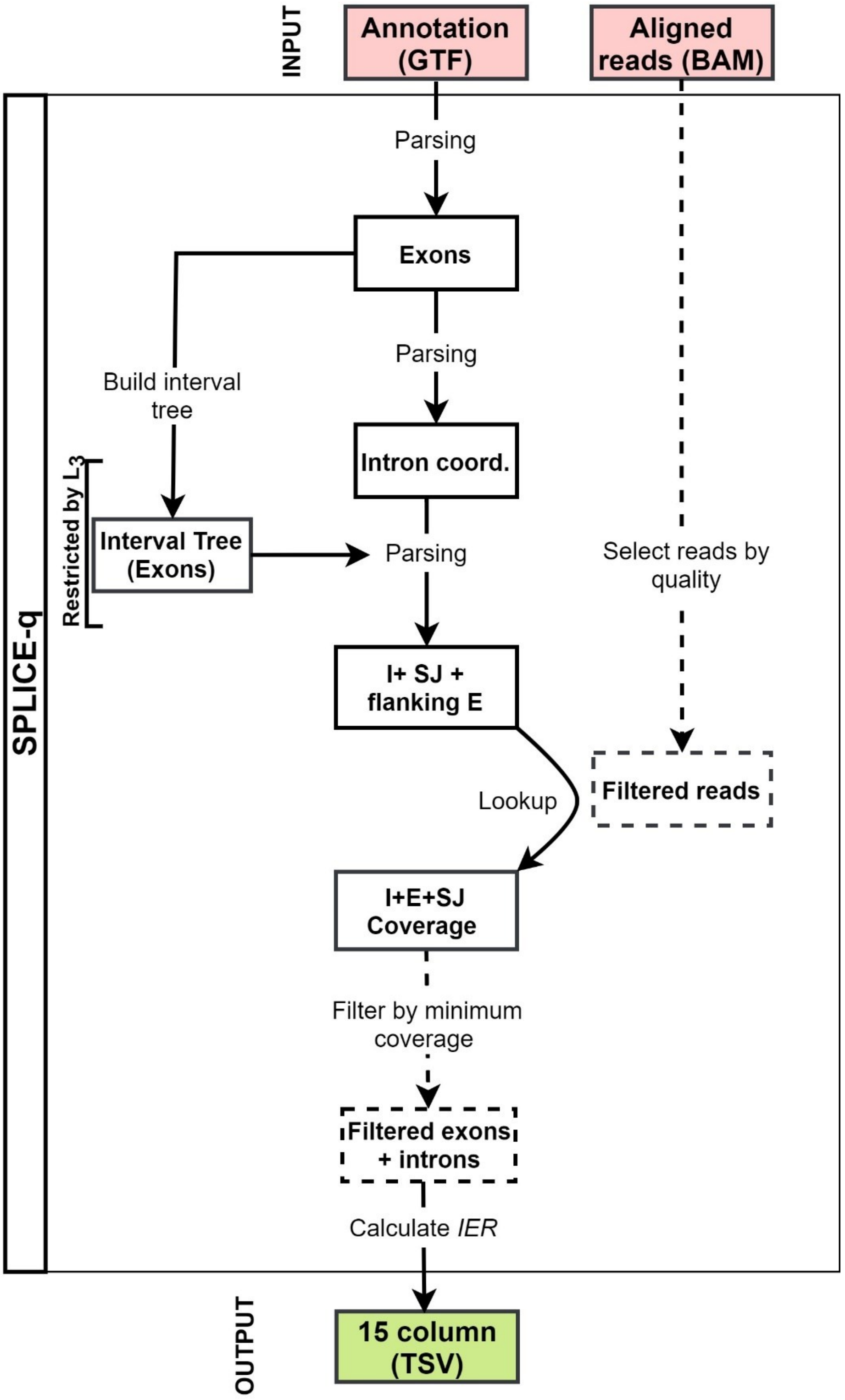

Fig. S1: SPLICE-q's inverse intron expression ratio (*IER*) workflow. Dashed lines indicate steps which depend on parameter settings. Solid lines represent the mandatory steps of the workflow. Boxes illustrate data types: input (red), intermediate data items (white) and output (green). I = intron; E= exon; SJ = splice junction; TSV = tab-separated values. Levels of restrictiveness: L_3 (Level 3).

a)

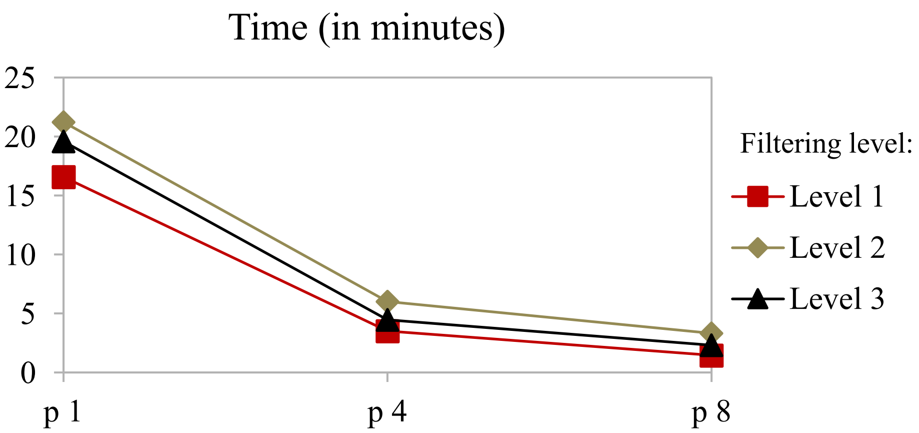

b)

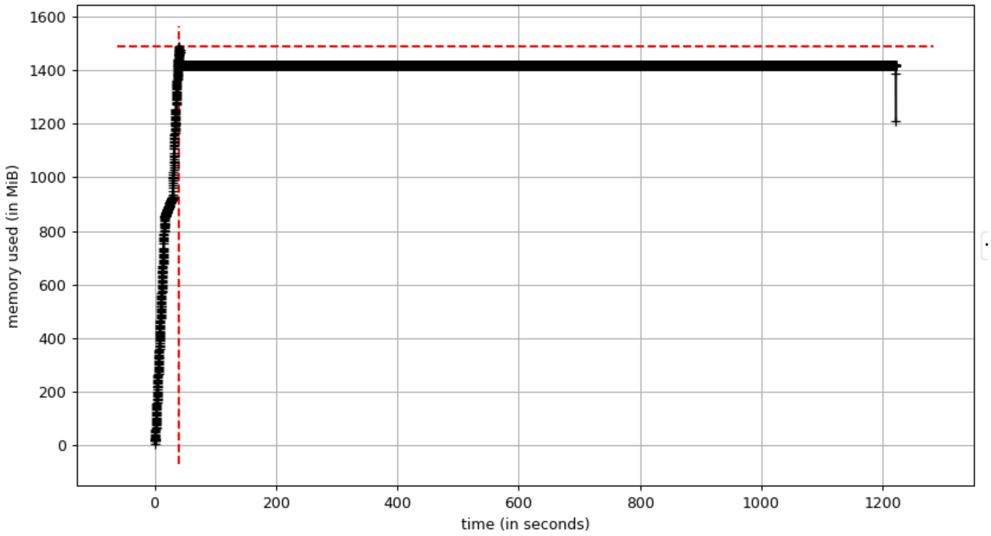

Fig. S2: **SPLICE-q's run time and memory usage**. **a)** Run time for approximately 100 million input reads mapped to the human genome (Linux, 64x AMD Opteron 6282 SE, 516GB). **b)** Memory usage for 1.4GB GTF. Time in seconds. p = Number of processes (*NProcesses*).

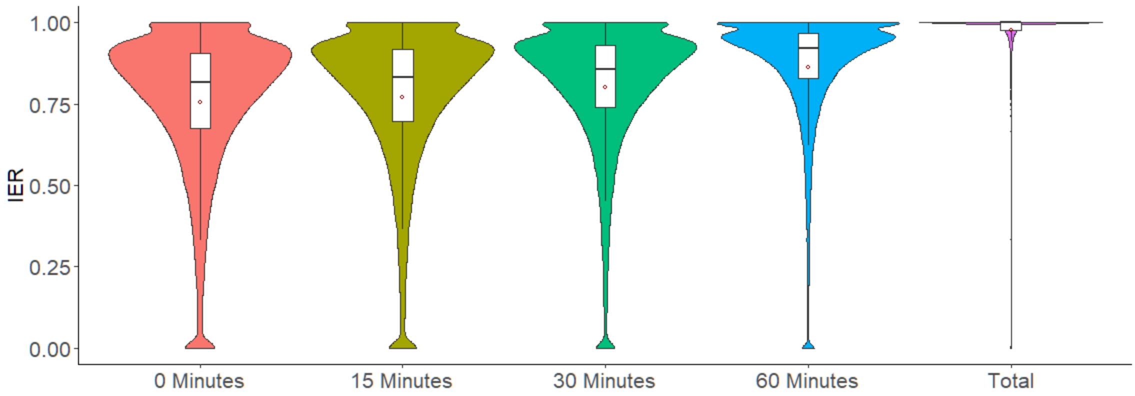

Fig. S3: Splicing kinetics using *IER*. Time-series nascent and steady-state (total) RNA-seq of labeled HEK293 cells.

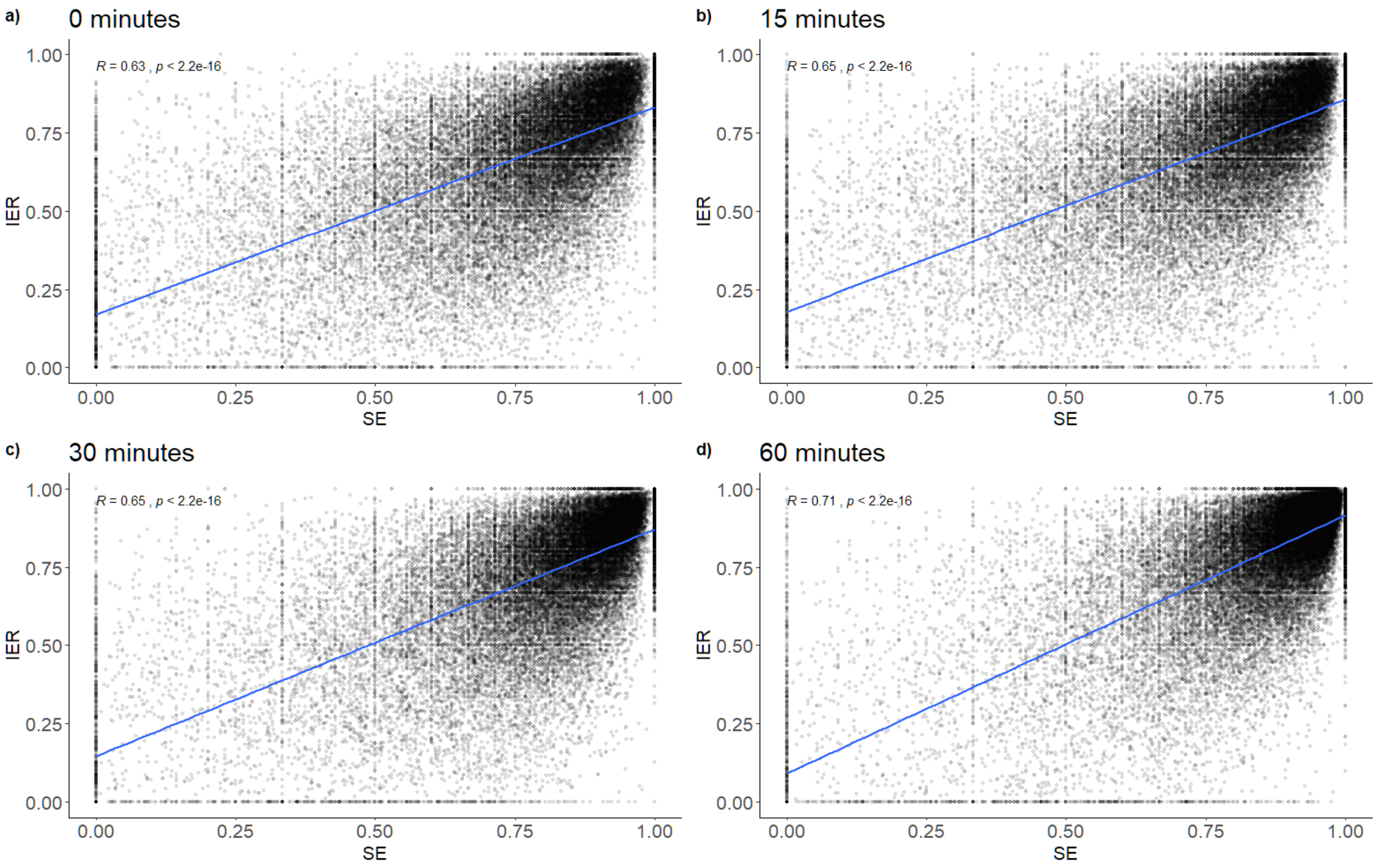

Fig. S4: Comparison of *SE* and *IER* scores. Time-series nascent RNA-seq of labeled HEK293 cells.

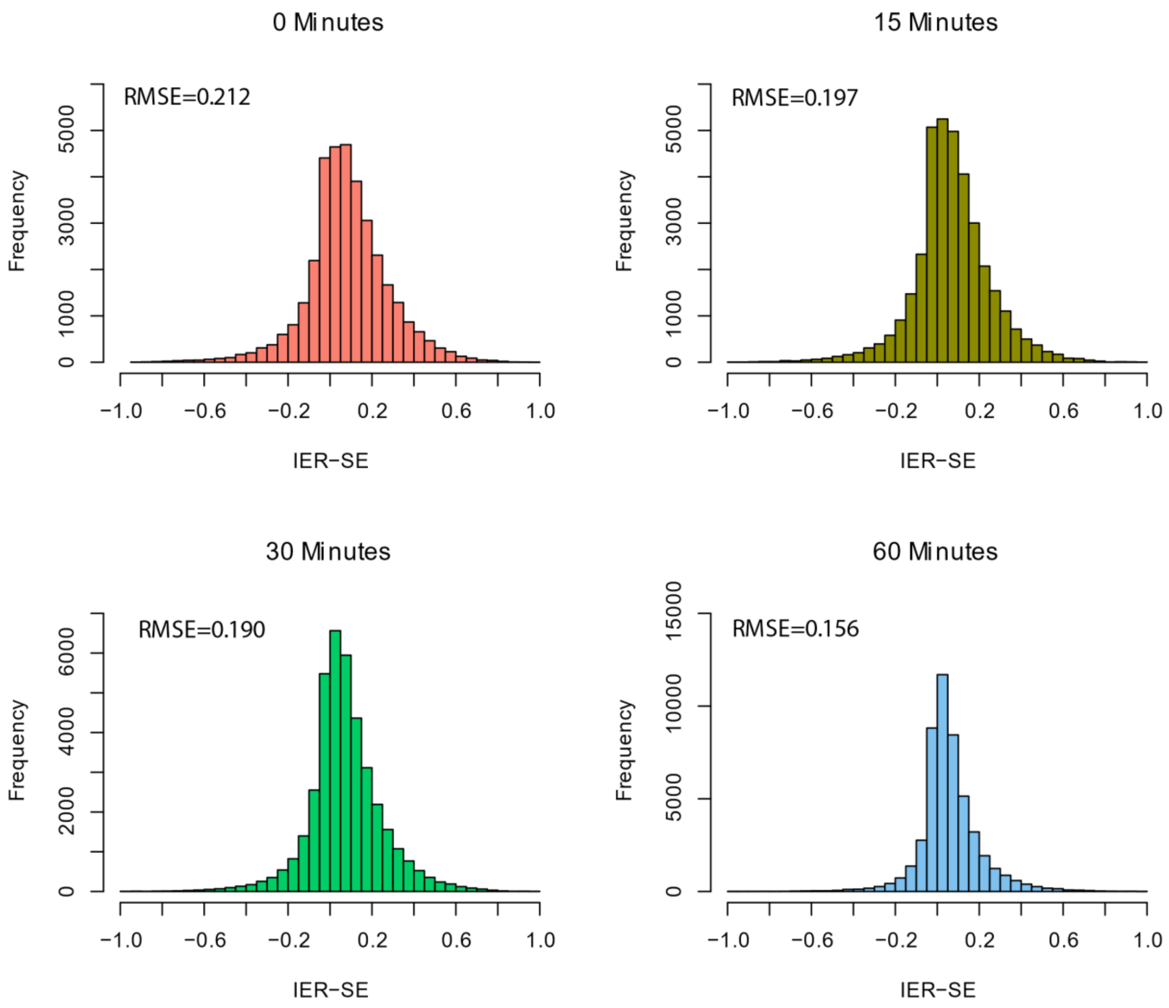

Fig. S5: Difference between *IER* and *SE* scores. Time-series nascent RNA-seq of labeled HEK293 cells. RMSE = Root-mean-square error. The distributions are slightly shifted towards positive values, indicating that *IER* scores are on average higher than *SE* scores. The closer the scores get to the maximum of 1 over the time course (compare to **Fig. 4a** in the paper and **Fig. S3** here), the smaller the differences get between *SE* and *IER*; this is also evident from the decreasing RMSE.

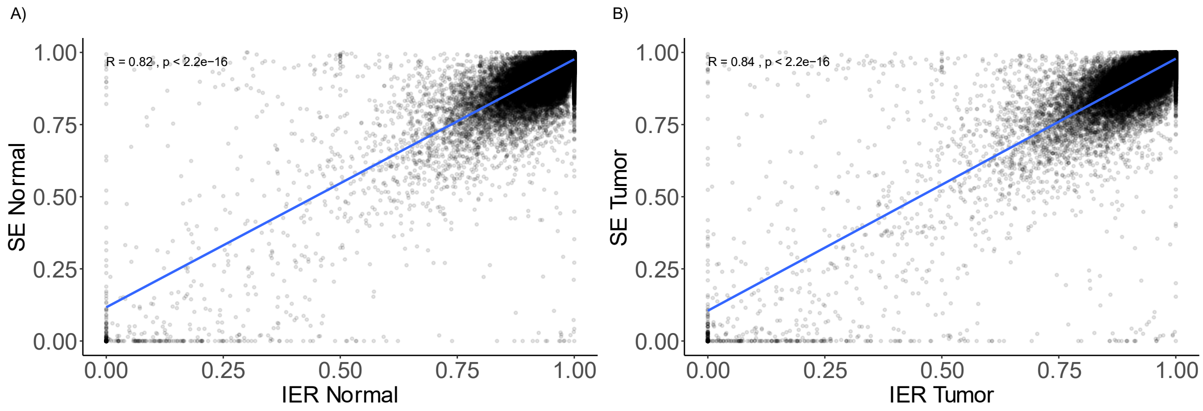

Fig. S6: Comparison of *SE* and *IER* scores for prostate tumor sample. Total RNA from prostate cancer tissue along with a matched normal control sample.

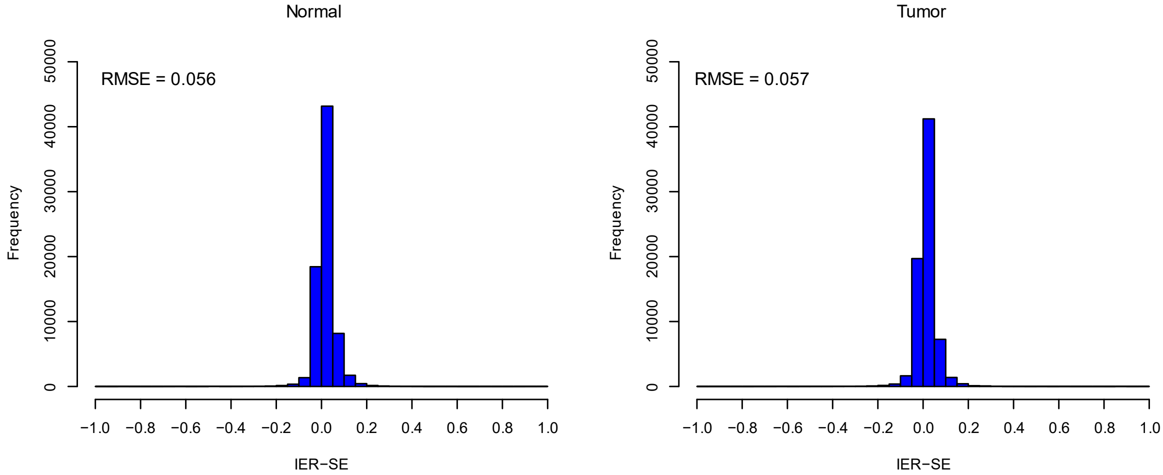

Fig. S7: Difference between *IER* and *SE* scores for prostate tumor sample. Total RNA from prostate cancer tissue along with a matched normal control sample. RMSE = Root-mean-square error.

Table S2: *SE* and *IER* of selected introns in control and tumor samples.

	Normal		Tumor	
	*SE**	*IER**	*SE**	*IER**
**PCA3** (chr9:76,782,833-76,783,704)	0.57	0.76	0.90	0.96
**RORβ** (chr9:74,630,368-74,634,630)	0.99	1.00	0.63	0.77
**RORβ** (chr9:74,634,773-74,642,413)	0.98	0.99	0.60	0.64
**SRPX2** (chrX:100,662,368-100,664,773)	0.59	0.79	0.90	0.85

**SE* and *IER* scores are averaged over the two replicates of the tumor sample and the normal control, respectively.

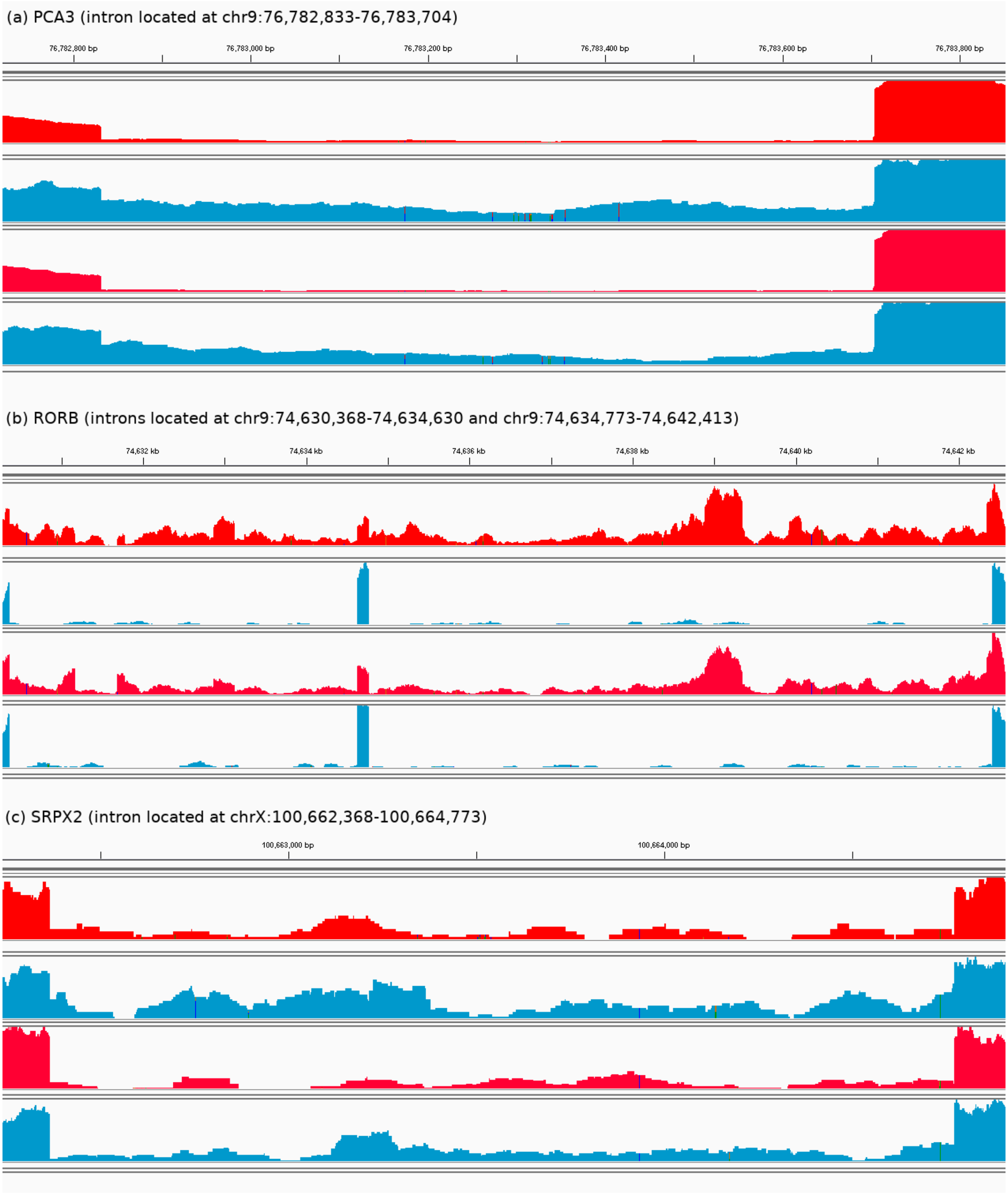

Fig. S8: Read coverage of selected introns in the prostate cancer and the normal control sample, showing the individual replicates. Tumor and normal samples are represented in red and blue, respectively. The introns shown are the same as those in Fig. 5 of the paper: **a)** PCA3, intron located at chr9:76,782,833-76,783,704; **b)** RORB, introns located at chr9:74,630,368-74,634,630 and at chr9:74,634,773-74,642,413; **c)** SRPX2, intron located at chrX:100,662,368-100,664,773.

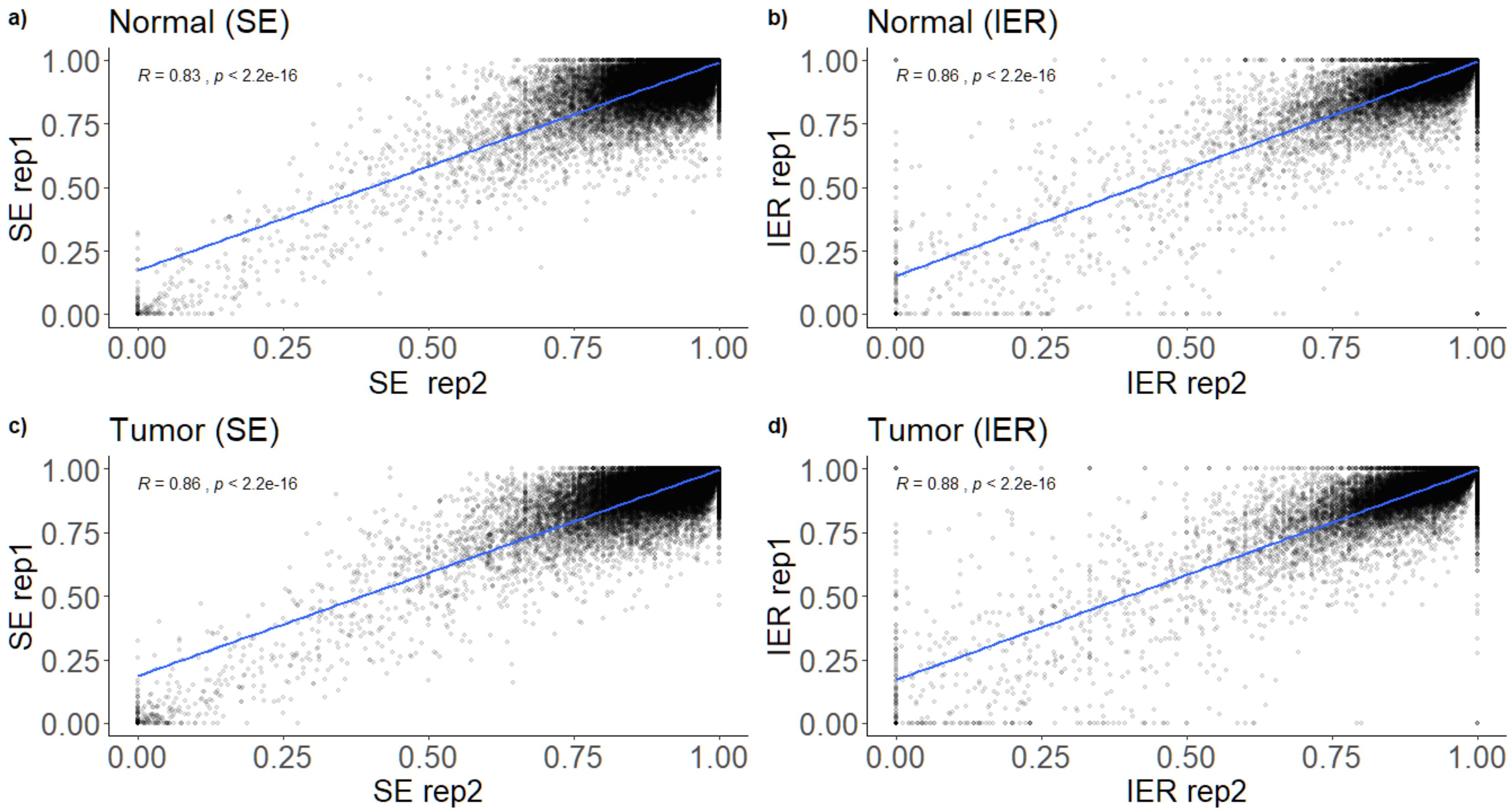

Fig. S9: Concordance of *SE* and *IER* scores across replicates. *SE* scores (a and c) and *IER* scores (b and d) are highly correlated across the two replicates of the prostate cancer tissue (c and d) and the normal control tissue (a and b). Note: some variation is to be expected, but the correlations shown here largely reflect the overall similarity of ρ = 0.90 (normal) and ρ = 0.92 (tumor) between the replicates (determined using DeepTools2.0 [5]).

Materials and Methods

BrU-chase, RNA-seq and read mapping

Human embryonic kidney cells (HEK293) cells were incubated for 15 minutes with 2mM of 5-bromouridine (BrU, pulse). Then, the cells were either collected immediately (0 minutes) or chased for 15, 30 and 60 minutes prior to RNA purification and selection of BrU-labeled RNA as described in [1]. The sequencing library was prepared with the TrueSeq Stranded Total RNA Kit (Illumina). Sequencing was performed in triplicate on the Illumina HiSeq 2500 platform to obtain an average of ~200 million reads per sample. Replicates read coverage are highly correlated with an average $\rho = 0.95$ which satisfies the ENCODE consortium recommendations for biological replicates [2]. The strand-specific reads were mapped to the human reference genome *GRCh38.p10* with STAR v2.7.1a [3] according to recommendations from the STAR manual 2.4.0.1. The genome index for STAR was built on the genome annotation from GENCODE v27[1]. An average of ~85% of the reads in all samples were uniquely mapped. The GEO [4] accession numbers for these sequencing data are GSE92565, GSE83561 and GSE84722.

[1]ftp://ftp.ebi.ac.uk/pub/databases/gencode/Gencode_human/release27/gencode.v27.annot ation.gtf.gz

Other datasets

The other datasets processed and analyzed are described below.

Table S3: Datasets used in the study.

Accession/ Reference	Genome/ Annotation	Description
GSE84722 [6]	GRCh38.p10/ gencode v27	Total RNA-seq of HEK293 cells. Sequenced on HiSeq2500.
GSE70378 [7]	Ensembl R64-1-1	*S. cerevisiae* labeled with 4tU labeling for 1.5, 2.5 and 5 minutes. Total RNA-seq also performed. All experiments were performed in triplicate. Sequenced on HiSeq2500.
GSE133626 [8]	GRCh38.p10/ gencode v27	Total RNA from fresh frozen prostate cancer tissue along with a matched normal control sample. Patient 15 of the dataset. Sequenced in duplicate on HiSeq2000.

Statistics and other methods

DeepTools2.0 [5] was used to assess genome-wide similarity of the sequencing replicates. All statistical tests were performed in R 3.6.1 (https://cran.r-project.org/). SPLICE-q's workflow Figures were generated with Drawio (available at https://github.com/jgraph/drawio).

References

1. Louloupi A, Ntini E, Conrad T, Ørom UAV. Transient N-6-Methyladenosine Transcriptome Sequencing Reveals a Regulatory Role of m6A in Splicing Efficiency. Cell Rep. 2018;23:3429–37. doi:10.1016/J.CELREP.2018.05.077.

2. The ENCODE Consortium. Standards, Guidelines and Best Practices for RNA-Seq. 2011. http://genome.ucsc.edu/ENCODE/protocols/dataStandards/ENCODE_RNAseq_Standards_V1.0.pdf. Accessed 6 Mar 2020.

3. Dobin A, Davis CA, Schlesinger F, Drenkow J, Zaleski C, Jha S, et al. STAR: ultrafast universal RNA-seq aligner. Bioinformatics. 2013;29:15–21. doi:10.1093/bioinformatics/bts635.

4. Barrett T, Wilhite SE, Ledoux P, Evangelista C, Kim IF, Tomashevsky M, et al. NCBI GEO: archive for functional genomics data sets—update. Nucleic Acids Res. 2012;41:D991–5. doi:10.1093/nar/gks1193.

5. Ramírez F, Ryan DP, Grüning B, Bhardwaj V, Kilpert F, Richter AS, et al. deepTools2: a next generation web server for deep-sequencing data analysis. Nucleic Acids Res. 2016;44:W160–5. doi:10.1093/nar/gkw257.

6. Mukherjee N, Calviello L, Hirsekorn A, de Pretis S, Pelizzola M, Ohler U. Integrative classification of human coding and noncoding genes through RNA metabolism profiles. Nat Struct Mol Biol. 2017;24:86–96. doi:10.1038/nsmb.3325.

7. Barrass JD, Reid JEA, Huang Y, Hector RD, Sanguinetti G, Beggs JD, et al. Transcriptome-wide RNA processing kinetics revealed using extremely short 4tU labeling. Genome Biol. 2015;16:282. doi:10.1186/s13059-015-0848-1.

8. Kumar A, Badredine A, Azzag K, Kasikçi Y, Ranty MLQ, Zaidi F, et al. Patient-matched analysis identifies deregulated networks in prostate cancer to guide personalized therapeutic intervention. bioRxiv. 2019;:695999. doi:10.1101/695999.
