# Supplementary material for: SPLICE-q: a Python tool for genome-wide quantification of splicing efficiency
Source: BMC Bioinformatics. 2021 Jul 15;22:368. doi: 10.1186/s12859-021-04282-6 (PMC8281633; doi:10.1186/s12859-021-04282-6)
Supplement: Supplementary file 2 — Additional file 2: Usage examples and performance evaluation for SPLICE-q. The file describes SPLICE-q’s basic usage and obtained output. It further provides information on SPLICE-q’s performance as compared to other tools and workflows, including a simulation of retention levels or reduced splicing efficiency. [file 12859_2021_4282_MOESM2_ESM.pdf]

Additional File 2

Usage examples and performance evaluation for SPLICE-q

SPLICE-q: a Python tool for genome-wide quantification of splicing efficiency

Verônica R Melo Costa[1,2,*], Julianus Pfeuffer[1,3,4], Annita Louloupi[5], Ulf A V Ørom[6], Rosario M Piro[7,*]

[1] Institute of Computer Science and Institute of Bioinformatics, Freie Universität Berlin, Berlin, Germany

[2] Department of Computational Molecular Biology, Max Planck Institute for Molecular Genetics, Berlin, Germany

[3] Department of Computer Science, Eberhard Karls Universität Tübingen, Tübingen, Germany

[4] Institute for Bioinformatics and Medical Informatics Tübingen, Eberhard Karls Universität Tübingen, Tübingen, Germany

[5] Berlin Institute for Medical Systems Biology, Max Delbrück Center for Molecular Medicine, Berlin, Germany

[6] Department of Molecular Biology and Genetics, Aarhus University, Aarhus, Denmark

[7] Dipartimento di Elettronica, Informazione e Bioingegneria, Politecnico di Milano, Milan, Italy

* Correspondence

veronica.melocosta@gmail.com

rosariomichael.piro@polimi.it

Contents

1. SPLICE-q's basic usage....3
2. Output....5
    2.1. Standard output....5
    2.2. *IER output*....5
3. Comparison of SPLICE-q to the Převorovský et al. workflow for quantification of splicing efficiency in yeast....7
    3.1. Comparision of splicing efficiency values....9
    3.2. Comparision of run time / performance....10
4. Comparision of SPLICE-q to IRFinder....11
    4.1. Comparision of run time / performance....12
5. Comparision of SPLICE-q to iREAD....14
    5.1. Comparision of run time / performance....15
6. PCA3, RORB and SPRX2 introns in IRFinder and iREAD....18
7. Simulation of intron retention levels or reduced splicing efficiency....19
References....22

List of Figures and Tables

Table S1: SPLICE-q's standard output (SE)....6
Table S2: SPLICE-q's IER output....6
Fig. S1: Comparison of splicing efficiency values....10
Table S3: Run-time measurements for SPLICE-q and Převorovský et al.'s workflow....11
Fig. S2: Comparison of SPLICE-q and IRFinder scores for nascent RNA-seq data....13
Fig. S3: Difference between SPLICE-q and IRFinder scores for nascent RNA-seq data....14
Fig. S4: Comparison of SPLICE-q and iREAD for nascent RNA-seq data....17
Fig. S5: Intron retention/splicing efficiency detection results with simulated RNA-seq data.21

1. SPLICE-q's basic usage

SPLICE-q (https://github.com/vrmelo/SPLICE-q) requires Python 3.6+ and can be easily installed via pip.

```
$ pip install SPLICE-q
```

To run SPLICE-q with default parameters, it requires aligned RNA-seq reads (i.e., a BAM file) and a genome annotation file in GTF format provided by GENCODE or Ensembl:

```
$ SPLICE-q.py -b <file.bam> -g <annotation.gtf>
```

By default, SPLICE-q prints results on the standard output, but an alternative output file name can be specified using the option `-o <outfile>`.

Below are further examples illustrating different use cases for SPLICE-q.

⇨ Chromosome names

SPLICE-q was developed to parse genomic features from the annotation file of most species. By default, it considers chromosomes chr1-720 (with or without the prefix "chr"), I-XVI, 2L, 2R, 3L, 3R, Z, W. If the BAM file contains different chromosome names or the user wishes to work with a particular subset of chromosomes, a list can be provided through the parameter `--ChromsList` or `-x`:

```
$ SPLICE-q.py -b <file.bam> -g <annotation.gtf> -x "1,2,3,4,X"
```

This is telling SPLICE-q to compute splicing efficiency measures for introns from chromosomes 1, 2, 3, 4 and X.

⇨ Coverage and mapping quality

By default, when computing *SE* scores, SPLICE-q works with uniquely mapped reads and a minimum of 10 reads mapping to **each** of the two splice junctions of an intron. These parameters can also be adjusted. For example:

```
$ SPLICE-q.py -b <file.bam> -g <annotation.gtf> -c 5 -r 1
```

`-c` 5 (or `--MinCoverage` 5) tells SPLICE-q to include introns whose splice junctions are covered by a minimum of 5 reads each.
`-r` 1 (or `--MinReadQuality` 1) tells SPLICE-q to include all reads with MAPQ >= 1.

⇨ **Multiprocessing**

Multiple concurrent processes can be used to minimize running times. The number of processes can be adjusted by the user through `-p` or `--NProcesses`:

```
$ SPLICE-q.py -b <file.bam> -g <annotation.gtf> -p 4
```

This option requires a BAM index file (.bai). SPLICE-q automatically checks if one is available and generates one in case it is not. By default, SPLICE-q instantiates four processes (`-p 4`).

⇨ **Overlap of genomic elements**

SPLICE-q is sensitive to the overlap of genomic elements. This means the tool takes into consideration when a genome shows overlapping features that can cause issues with a correct assignment of reads to specific introns or exons. For example, for introns which overlap exons of other genes can be filtered out. The different levels of restrictiveness for **strand-specific** filtering are:

- **Level 1**: keeps all introns in the genome regardless of overlaps with other genomic elements.
- **Level 2**: selects only introns whose splice junctions do not overlap any exon of a different gene.
- **Level 3**: selects only introns that do not overlap with any exon of the same or a different gene. This is the default option.

Users wishing to select levels 1 or 2 for their analysis can simply use the parameter `--FilterLevel` or `-f`. For example:

```
$ SPLICE-q.py -b <file.bam> -g <annotation.gtf> -f 2
```

This will run SPLICE-q on **Level 2**.

⇨ **Reverse Intron Expression Ratio (*IER*)**

As an alternative measure for splicing efficiency, SPLICE-q computes an inverse intron expression ratio (*IER*), which compares the introns' expression levels with those of their flanking exons. *IER* can be quantified with the options `-e` or `--IERatio`:

```
$ SPLICE-q -b <file.bam> -g <annotation.gtf> -e
```

This option requires filtering level `-f 3` (default). The other parameters can be adjusted without restrictions when computing *IER* (except `--MinCoverage`/`-c`, i.e., the minimum coverage at splice junctions, which is ignored)

2. Output

2.1 Standard output

The standard output is a 14-column tab-separated values (TSV) file containing: chromosome, strand, gene ID, transcript ID, intron number (within the gene), SJ5' start position, SJ5' end position, SJ5' split read count, SJ5' unsplit read count, SJ3' start position, SJ3' end position, SJ3' split read count, SJ3' unsplit read count, *SE* score. See Table S1 for an example.

2.2 *IER* output

When running with the inverse intron expression ratio option, the program outputs a 15-column TSV consisting of chromosome, intron start position, intron end position, strand, intron number, gene ID, transcript ID, 5' exon median coverage, SJ5' split read count, SJ5' unsplit read count, intron median coverage, SJ3' split read count, SJ3' unsplit read count, 3' exon median coverage and *IER*. See Table S2 for an example.

Table S1: SPLICE-q's standard output (*SE*)

chr	strand	gene_ID	transcript_ID	intron_ID	sj5start	sj5end	sj5_cov_nonsplit	sj5_cov_split	sj3start	sj3end	sj3_cov_nonsplit	sj3_cov_split	SE
1	+	ENSG00000000460.16	ENST00000413811.3	3	169800971	69800972	22	9	169802620	169802621	25	4	0.7833
1	+	ENSG00000000460.16	ENST00000413811.3	4	169802725	169802726	16	4	169803168	169803169	16	2	0.8421
1	+	ENSG00000000460.16	ENST00000413811.3	9	169823472	169823473	9	10	169827050	169827051	9	3	0.5806
17	-	ENSG00000108654.13	ENST00000581237.2	2	64500548	64500549	63	320	64500326	64500327	66	166	0.2097

Table S2: SPLICE-q's *IER* output

chr	IStart	IEnd	strand	intron_ID	gene_ID	transcript_ID	exon5_cov	sj5_cov_split	sj5_cov_nonsplit	intron_cov	sj3_cov_split	sj3_cov_nonsplit	exon3_cov	IER
1	169800971	169802620	+	3	ENSG00000000460.16	ENST00000413811.3	17.0	22	9	2.0	25	4	22.0	0.8974
1	169802725	169803168	+	4	ENSG00000000460.16	ENST00000413811.3	22.0	16	4	1.0	16	2	10.0	0.9375
1	169823472	169827050	+	9	ENSG00000000460.16	ENST00000413811.3	21.0	9	10	2.0	9	3	11.0	0.8750
17	64500326	64500548	-	2	ENSG00000108654.13	ENST00000581237.2	387.0	63	320	193.0	66	166	148.0	0.2785

3. Comparison of SPLICE-q to the Převorovský et al. workflow for quantification of splicing efficiency in yeast

Převorovský et al. [1] presented a workflow for genome-wide determination of splicing efficiency in yeast. The workflow starts from FASTQ files and first performs quality control, then read mapping and finally the quantification of splicing efficiency for each splice junction separately. This workflow relies on numerous pre-installed open-source tools (FastQC, HISAT2, Samtools, Regtools and Bedtools) and custom shell and R scripts and was explicitly developed for *Saccharomyces cerevisiae* (the file names of the reference genome and annotation file are hard-coded and any change requires to adapt the source code of the scripts accordingly). The set of scripts can be downloaded from the supplementary material of the original paper by Převorovský et al. [1] and needs to be thoroughly adapted for RNA-seq datasets other than those used by the original authors, requiring thus familiarity with shell and R scripting. The supplementary material, provided by the authors, includes also an alternative version of the workflow for processing RNA-seq data from *Schizosaccharomyces pombe*.

Apart from the installation of the above mentioned tools, the time used to manually adapt Převorovský et al.'s workflow was considerable. Furthermore, some of the parameters of the pre-installed tools used by the workflow's script were outdated and needed to be corrected in the code. The following changes had to be made before running the workflow for the 4tU-seq yeast data and the prostate cancer data used in our paper:

1. In the main shell script of the workflow (*workflow.sh*):
   a) We commented out FastQ file merges which were required for the data originally used by the authors for their publication, e.g.:
      - `cat ./FASTQ/1-BY4741-b_1.fq.gz ./FASTQ/13-BY4741-_1.fq.gz > ./FASTQ/WT_1.fastq.gz`

   a) We updated the use of "`regtools junctions extract`" by *workflow.sh*, due to a change of the regtools command line interface since the publication of the workflow:
      - previous version:

        `-i ${min_intron} -I ${max_intron}`

- now:

  `-m ${min_intron} -M ${max_intron} -s 1`

  where "`${min_intron}`" is a minimum intron length of 20 and "`${max_intron}`" is a maximum intron length of 10000, both hard-coded in the *workflow.sh* script, and "`-s 1`" stands for "first-strand/RF", indicating strand-specific RNA-seq data where the first read (or the only read in case of SE) is from the opposite strand (alternatively, "`-s 2`" can be used for second-strand data and "`-s 0`" for unstranded data).

2. In the R script which identifies intron-exon boundaries and counts junction reads (*junctions.R*):

   a) The script was written for (and works well with) yeast data, but introduces problems for human data because it writes large chromosomal coordinates in scientific notation in output files (e.g., "1.4e+07" instead of "14000000"). This causes errors in downstream analysis steps. To circumvent the problem, the following R command can be added to the beginning of the script:

   - `options(scipen=999)`

3. In the R script which computes the splicing efficiencies (*efficiency.R*):

   a) We changed hard-coded sample names, e.g.:

   - `comparisons <- t(matrix(c('14-prp45-_1', '13-BY4741-_1', '7-AVY17-b_1', '1-BY4741-b_1', 'prp45_1', 'WT_1'), nrow = 2, ncol = 3))`

   b) If desired, the user has to change the hard-coded default threshold for the minimum number of reads located at each splice junction of an intron:

   - `threshold.counts <- 5`

To run the workflow on data from other species, also the names of the reference genome and the annotation file need to be manually changed in the workflow scripts.

The most important manual modifications to the workflow, however, that a potential user should keep in mind, regard the strandness of the RNA-seq data to be analyzed. The strandness needs to be correctly specified in the function call of the HISAT2 read aligner, the function call of regtools (see above), and for bedtools multicov. From the description of these necessary modifications, it should be clear that running the workflow correctly is a significant challenge for users unfamiliar with shell scripting and R programming.

To produce a comparable output with SPLICE-q, we adjusted the parameters to match the gene annotation and cutoffs hard-coded in Převorovský et al.'s workflow as follows:

```
$ SPLICE-q.py -b <in.bam> -g Saccharomyces_cerevisiae.R64-
1-1.84.gtf -f 1 -l 20 -c 5
```

Thus, we ignore overlaps of genomic elements, consider only introns with a minimum length of 20 bp and include introns whose splice junctions are covered by a minimum of 5 reads each. While SPLICE-q provides a single output containing all the information used for the splicing efficiency (*SE*) quantification, allowing the user to easily access information such as read counts and coordinates, the Převorovský et al.'s workflow outputs 12 files distributed in 3 folders (not shown here).

3.1 Comparison of splicing efficiency values

The workflow [1] quantifies the splicing efficiencies for each intron's 5' splice site and 3' splice site separately as the number of "*transreads*", i.e., split reads spanning from exon to exon divided by the number of reads covering the first or last base of the intron. To compare the output to the one obtained for SPLICE-q, we used the 4tU-seq data from *S. cerevisiae* (time point 1.5 minutes) [2] used in our paper.

Although the authors call their metric "splicing efficiency", the values are not limited to a range from 0 to 1. In fact, some of the outliers had extremely high values ("splicing efficiency" > 5000) making the workflow's scores not directly comparable to SPLICE-q's *SE*. Hence, we limit our analysis to correlations between the two measurements, but ignore absolute differences between the scores obtained by the two methods.

While there are notable differences between SPLICE-q and Převorovský et al.'s workflow, overall, the splicing efficiencies for individual introns provided by the two measures are strongly correlated (**Fig. S1**).

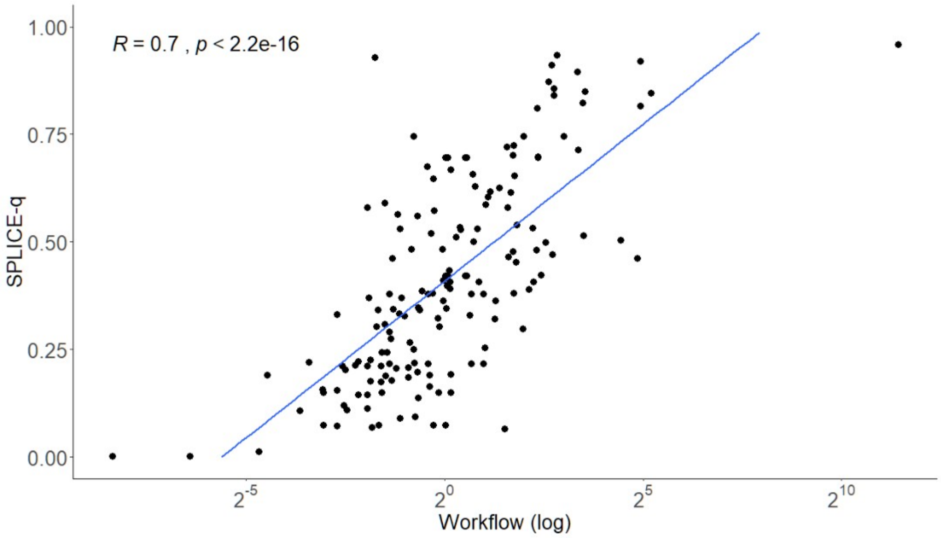

Fig. S1: Comparison of splicing efficiency values. *SE* (SPLICE-q, y-axis) and the log-transformed splicing measure computed by Převorovský et al.'s workflow (x-axis) for individual introns, computed from 4tU-seq data from *S. cerevisiae* (time point 1.5 minutes) [2]. Pearson correlation coefficient: 0.699, Spearman rank correlation: 0.703 (p < 2.2e-16 for both).

3.2 Comparison of run times / performance

We tested both SPLICE-q and Převorovský et al.'s workflow on a Dell Precision Mobile Workstation (laptop with Linux, Intel Core i7-9850H, 32 GB physical memory).

We ran Převorovský et al.'s full workflow but measured time consumption for the quality control plus read mapping and the following splicing efficiency analysis separately. For SPLICE-q, which does not include a read mapping step, we used the BAM files produced by Převorovský et al.'s workflow and performed only the splicing efficiency analysis. Since only quality control and read mapping are run on multiple cores (four cores, hard-coded) in Převorovský et al.'s workflow, while splicing efficiencies are computed on a single core, we also used one core for SPLICE-q. For further comparison, we additionally ran SPLICE-q also on 4 cores.

We first determined the run times for one single sample of 4tU-seq data from *S. cerevisiae* (time point 1.5 minutes) [2]. Then, we tested a more demanding dataset: a total of four samples of the prostate cancer patient analyzed in our paper (two replicates for the tumor and two replicates for the normal control; patient 15 of ref. [3]). Since Převorovský et al.'s workflow was developed for single-end RNA-seq data, we considered only the FASTQ files

for read 1 (R1) [2]. Then, we tested a more demanding dataset: a total of four samples of the prostate cancer patient analyzed in our paper (two replicates for the tumor and two replicates for the normal control; patient 15 of ref. [3]). Since Převorovský et al.'s workflow was developed for single-end RNA-seq data, we considered only the FASTQ files for read 1 (R1).

As shown in Table S3, for a small dataset and a species with few introns (yeast), Převorovský et al.'s workflow is slightly faster than SPLICE-q which, however, yields comparable results. For a larger dataset and a species with a significantly higher number of introns (human), SPLICE-q has a decisive advantage with significantly improved run times.

Especially for larger datasets, running SPLICE-q on multiple cores can further speed up the computation of splicing efficiencies. For the smaller dataset, most of the time is spent reading input data (which is done on a single core), such that the computation of splicing efficiencies on 4 cores can shorten run times only slightly.

Table S3: Run-time measurements for SPLICE-q and Převorovský et al.'s workflow

Data	Total number of mapped reads analyzed	Number of introns in the GTF file (genome annotation)	Quality control (FastQC) and read mapping (HISAT2) [Workflow]	Splicing efficiency computation		
				Workflow (1 core)	SPLICE-q (1 core)	SPLICE-q (up to 4 cores)
4tU-seq data (*S. cerevisiae*; time point 1.5 minutes of [2])	36,386,555	427	65.5 min	1.97 min	2.66 min	2.09 min
Prostate cancer (*H. sapiens*; patient 15 of [3])	152,603,346	236,373	100.5 min	125.3 min	17.57 min	6.53 min

4. Comparison of SPLICE-q to IRFinder

IRFinder [4] was designed to detect intron retention from mRNA-seq data. The end-to-end analysis ("FastQ" mode) includes the alignment of sequencing reads using STAR [1], quality control and the quantification of intron retention levels. IRFinder can also be used to compare intron retention levels between multiple samples. Each step of the workflow is provided by a single module that can be used independently, and the user can easily set up the workflow starting from pre-aligned reads (unsorted BAM files) as input ("BAM" mode). The quality control filter issues warnings when the data is not suitable for intron retention detection, i.e., data incorrectly labeled as RNA-seq experiments or data from non poly(A)-enriched RNA-seq.

IRFinder computes a score called *IRratio* as the intron's average base coverage (number of reads divided by length) divided by said intron coverage plus the base coverage of the spliced transcript (using as a proxy the higher value of the number of split reads mapping to the 5' splice junction and the number of split reads mapping to the 3' splice junction of the intron). [Note: while the IRFinder paper [4] states that the sum of the two read counts are used, which makes much less sense from a biological point of view, the published source code of version 1.3.1 actually uses the maximum of the two read counts, as described here]. Intronic parts that overlap with other known genomic features (e.g., intronic snoRNAs) are excluded from the calculation of per-base intron coverages. Since IRFinder is not available as a stand-alone package, the dependencies need to be manually installed by the user (Perl, STAR, Samtools and Bedtools). Furthermore, the module for intron retention quantification of the tool ("BAM" mode) does not offer filtering options as parameters. The output has to be filtered manually (e.g., regarding minimum read depth of overlap of intron-exon boundaries with other genomic elements).

We analyzed two time points of the HEK293 nascent RNA-seq data (0 minutes and 60 minutes) to compare SPLICE-q and IRFinder (version 1.3.1). Although there are differences between the results of both tools, overall, the splicing scores for individual introns provided by them (*SE* and *IER* for SPLICE-q and the *IRratio* for IRFinder) show a high negative correlation. See Fig. S2 for scatterplots and Fig. S3 for the distributions of differences between the measures; for the latter, we use the inverse of the *IRratio* score (1-*IRratio*) as a direct comparison to *SE* and *IER*.

4.1 Comparison of run times / performance

We tested IRFinder (version 1.3.1) on a Dell Precision Mobile Workstation (laptop with Linux, Intel Core i7-9850H, 32 GB physical memory) using the prostate tumor data described in our paper. Since IRFinder cannot run on multiple cores (no parallelization), we also ran SPLICE-q on a single core. Even running on a single core, SPLICE-q has improved run times (17.6 minutes as opposed to 28.5 minutes of elapsed time for IRFinder) and requires less memory (1.33 GB as opposed to 3.28 GB peak memory usage). As previously mentioned, running SPLICE-q on multiple cores can further speed up the computation of splicing efficiencies

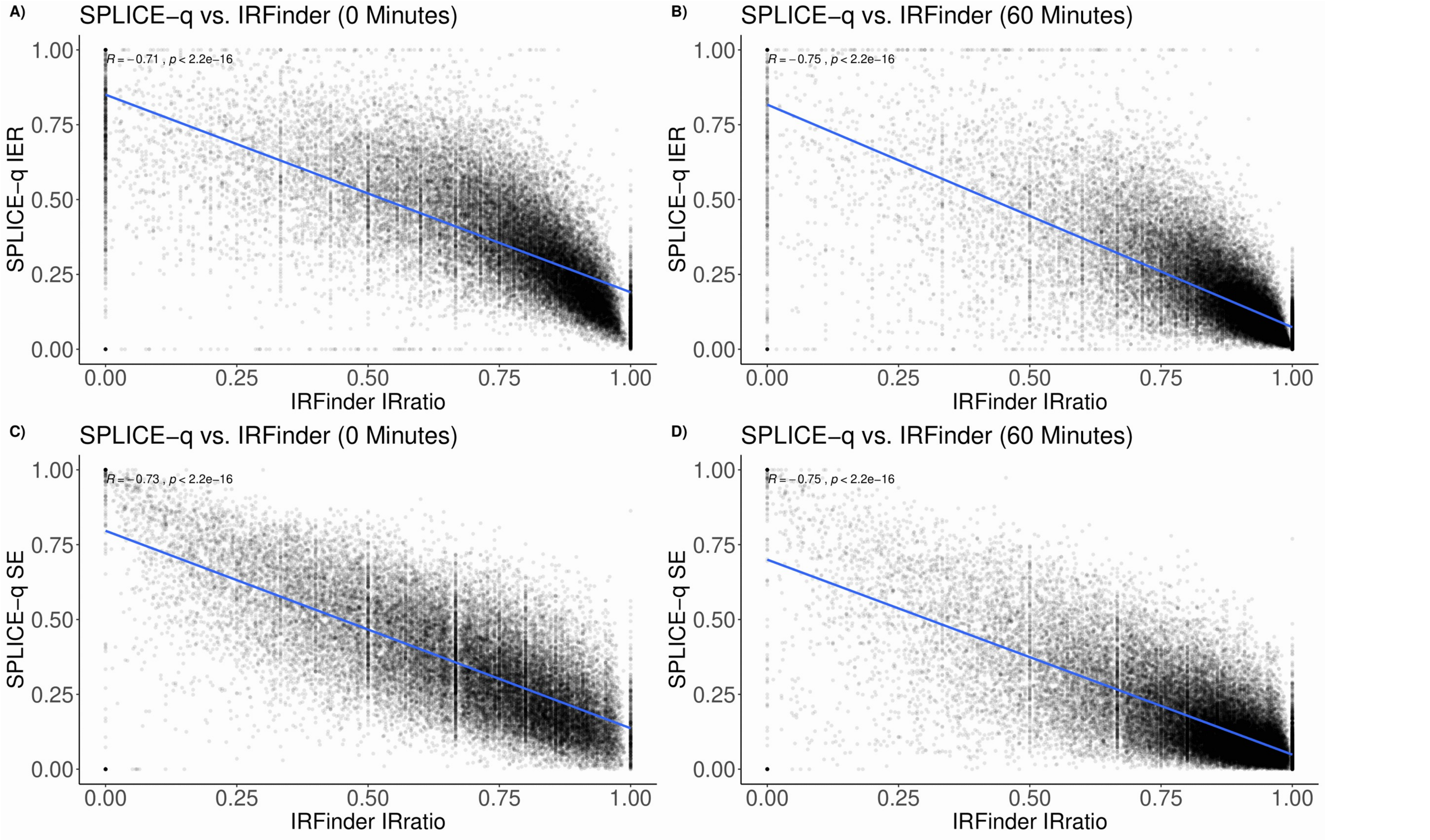

Fig. S2: Comparison of SPLICE-q and IRFinder scores for nascent RNA-seq data. HEK293 nascent RNA-seq data (0 minutes and 60 minutes). Only introns satisfying SPLICE-q's default filtering criteria in all samples were kept.

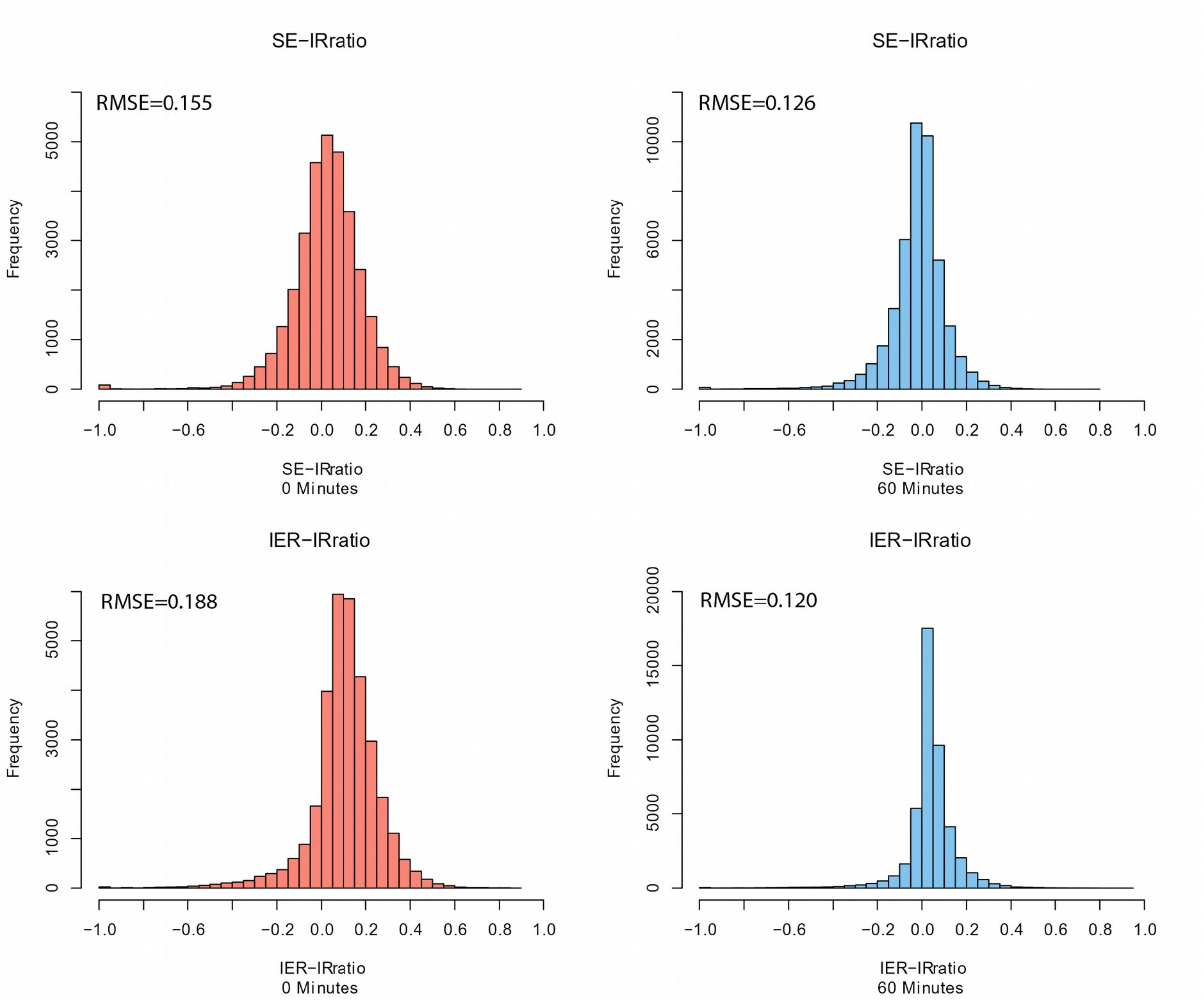

Fig. S3: Difference between SPLICE-q and IRFinder scores for nascent RNA-seq data. HEK293 nascent RNA-seq data (0 minutes and 60 minutes). Only introns satisfying SPLICE-q's default filtering criteria in all samples were kept. For comparison, we use the inverse of IRFinder's *IRratio* score (1-*IRratio*). RMSE = Root-mean-square error.

5. Comparison of SPLICE-q to iREAD

iREAD (intron REtention Analysis and Detector) is another tool developed to detect intron retention events from RNA-seq data [5]. The iREAD algorithm takes aligned reads (BAM file sorted by coordinate and its index) together with a list of introns that do not overlap with other genomic elements in BED format. iREAD provides these intron coordinates for human, mice and *D. Melanogaster* for download on their website (https://github.com/genemine/iread). However, users working with different species or annotation versions are required to independently calculate the intron annotations, which brings some inconveniences, specially for users not familiar with parsing genomic features.

Furthermore, iREAD is not available as a stand-alone package and the dependencies need to be manually installed (BEDOPS, Samtools and the PERL module Parallel::ForkManager).

iREAD uses intron expression levels and splice junction reads to detect retained introns. The tool computes an entropy score that assesses the "flatness" of the distribution of intronic reads quantified by dividing each intron into eight bins in which the number of reads is counted and normalized. Then, the entropy is calculated as described in ref. [5].

The final, binary decision of whether a specific intron is to be considered as retained or not depends on arbitrary thresholds for the total number of intronic reads (default: ≥20), the number of split reads (default: ≥1), the intron expression level (FPKM; default: ≥3) and the entropy score (default: ≥0.9).

To compare SPLICE-q and iREAD (version 0.8.9), we analyzed two timepoints of the HEK293 nascent RNA-seq data (0 minutes and 60 minutes), using for both the gene annotation (or derived intron annotation) from GENCODE v27. Since the binary output (intron retained or not) of iREAD is not directly comparable to neither *SE* scores nor *IER* scores, we binned *SE* scores or *IER* scores into bins of width 0.1 and verified what fraction of corresponding introns is declared to be retained by iREAD with default settings (see Fig. S4). While there are some notable exceptions, as expected introns with lower *SE* or *IER* scores tend to be more frequently considered as retained by iREAD, especially for the nascent RNA-seq data at 0 minutes. Yet, the fraction of introns identified by iREAD always remains rather low.

5.1 Comparison of run times / performance

Although iREAD optionally allows to specify the maximum available memory as a command line option, unfortunately not all of the various Python, Perl and Bash scripts which compose the tool do actually accept this parameter. Especially for very large BAM files, the memory requirement can easily exceed the 32 GB available on the Dell Precision Mobile Workstation (laptop with Linux, Intel Core i7-9850H, 32 GB physical memory) on which we tested the other tools. We therefore compared the performance of SPLICE-q and iREAD (version 0.8.9) on a computing system with 2x Dual-Core AMD Opteron 8220 processors (16 available cores) and 264 GB of physical memory. For the largest of the BAM files from the HEK293 nascent RNA-seq dataset (15.88 GB file size), iREAD with default settings ran for 3.31 hours and had a peak memory usage of 166.6 GB, although the command line parameter

was set for a maximum of 32 GB. For one of the prostate cancer replicates (BAM file size 9.97 GB), iREAD required 1.08 hours with a peak memory usage of 35.5 GB. For the same BAM files, SPLICE-q with default settings (except for using 14 cores because iREAD by default uses up to *N* cores, where *N* is the maximum number minus 2; here 16-2=14). SPLICE-q completed processing the two BAM files in only 18.9 minutes (1.32 GB peak memory usage) and 13.8 minutes (1.33 GB peak memory usage), respectively.

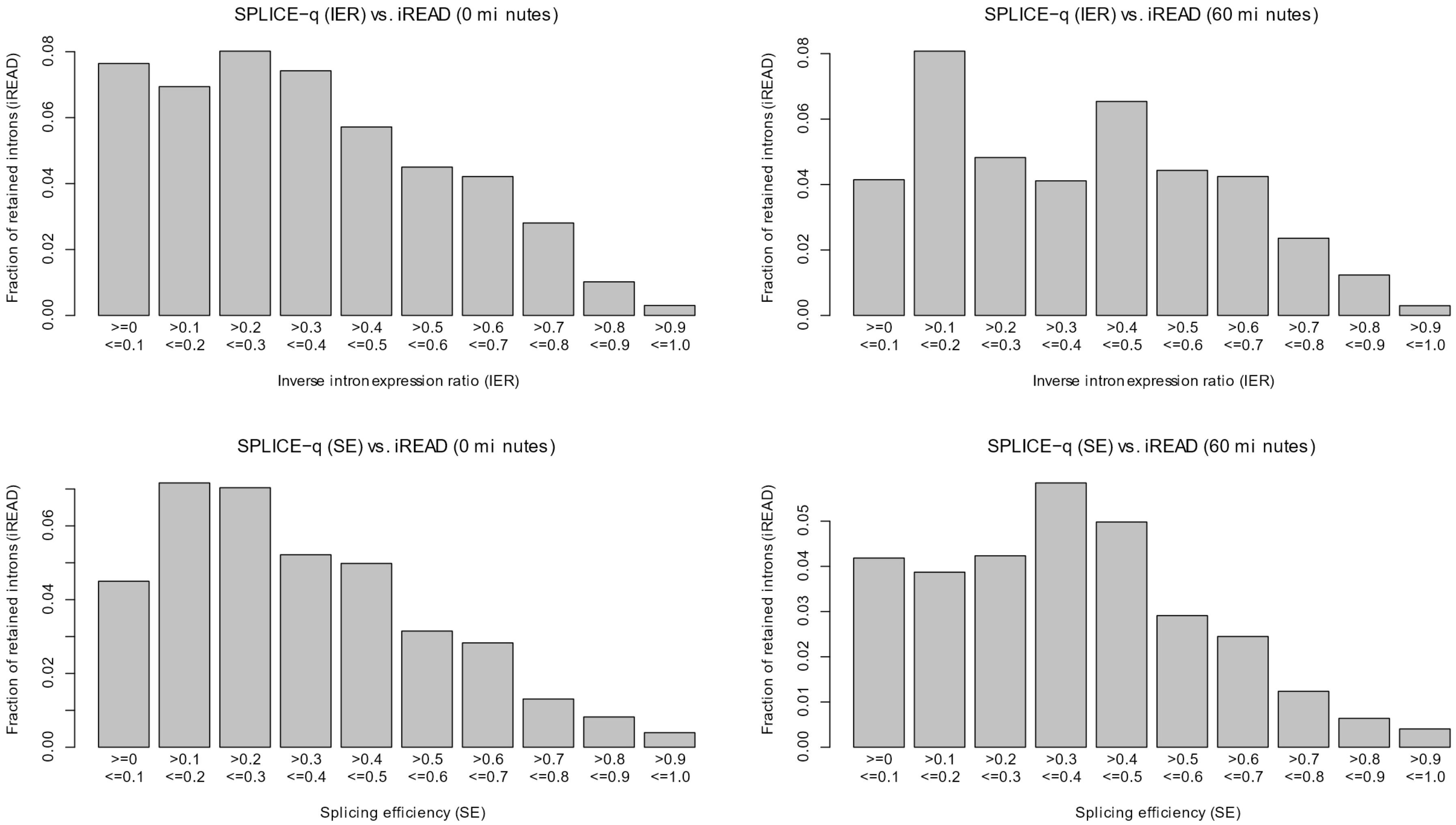

Fig. S4: Comparison of SPLICE-q and iREAD for nascent RNA-seq data. HEK293 nascent RNA-seq data (0 minutes and 60 minutes). Only introns satisfying SPLICE-q's default filtering criteria in all samples were kept. The plots show the fractions of intron retention events detected by iREAD (y-axes) among the introns falling into specific bins of *SE* or *IER* scores as determined by SPLICE-q (x-axes).

6. PCA3, RORB and SPRX2 introns in IRFinder and iREAD

We checked the results we obtained for iREAD and IRFinder (default settings) for the four introns shown Fig. 5 of our paper (and Fig. S8 in Additional file 1), using the same gene annotation (GENCODE v27) we had used for SPLICE-q:

- PCA3, intron located at chr9:76,782,833-76,783,704:
  - IRFinder: despite having used the same gene annotation for building the IRFinder index, we found no results for PCA3 in the IRFinder output.
  - iREAD detected no intronic fragments and junction reads for both the normal and the tumor replicates, so it considered the intron as not retained. This is somewhat surprising given that Fig. 5 clearly shows intronic reads, especially in the normal control.
- RORB, intron located at chr9:74,630,368-74,634,630
  - IRFinder: normal tissue replicates: *IRratio* of 0.00837 and 0.00784 (average: 0.00811); tumor tissue replicates: *IRratio* of 0.02771 and 0.02545 (average: 0.02658). This would correspond roughly to an *SE* of 1-0.00811=0.99189 for the normal tissue and an *SE* of about 1-0.02658=0.97342 for the tumor. The differences seem marginal, so IRFinder would probably not have detected the change in splicing efficiency we can actually see in Fig. 5.
  - iREAD detected no intronic fragments and junction reads for both the normal and the tumor replicates, so it considered the intron as not retained. This is somewhat surprising given that Fig. 5 clearly shows intronic reads, especially in the tumor.
- RORB, intron located at chr9:74,634,773-74,642,413
  - IRFinder: as for the other intron of RORB, IRFinder would unlikely have detected any difference: *IRratio*s are 0.01062 and 0.00657 for the normal replicates and 0.02419 and 0.01436 for the tumor replicates.
  - iREAD detected no intronic fragments and junction reads for both the normal and the tumor replicates, so it considered the intron as not retained. This is somewhat surprising given that Fig. 5 clearly shows intronic reads, especially in the tumor.
- SRPX2, intron located at chrX:100,662,368-100,664,773

- IRFinder: like for the RORB introns, IRFinder would not have detected any meaningful difference (*IRratio*s for normal: 0.02080 and 0.00805; for tumor: 0.01474 and 0.01308).
- iREAD detected both intronic fragments and junction reads for the normal and the tumor replicates, but still considered the introns as not retained, mostly due to low entropy scores, it thus considers the intronic expression not to be uniform enough to call an intron retention event.

Thus, none of the events shown in Fig. 5 of our paper (and Fig. S8 in Additional file 1) would have been detected by IRFinder or iREAD.

7. Simulation of intron retention levels or reduced splicing efficiency

Since to our best knowledge, there is no gold standard RNA-seq data set containing a set of experimentally verified intron retention events, in order to be able to directly compare the capabilities of SPLICE-q, IRFinder and iREAD to detect such events, we simulated a set of RNA-seq samples and applied the methods to verify whether they were able to identify different intron retention levels (or the opposite, i.e., splicing efficiency levels).

In order to reduce the computational time required to simulate multiple data sets with different characteristics, especially due to the need to also simulate intronic reads, we determined a small set of 527 genes with a total of 1213 introns from the GENCODE v27 gene annotation applying the following filters:

1. We excluded all genes which overlap with other genes on the same strand or which do not lie on one of the regular human chromosomes (1-22, X, Y, M), i.e., we excluded all decoy sequences.
2. We excluded all genes which do not code for proteins or do not contain any introns.
3. For simplified handling and further reduction of the number of genes, we limited the analysis to genes on the forward strand of the reference genome.
4. Genes were kept only if all their introns have lengths between $2*N$ and $20*N$, where N is the simulated read length.

We used RNASeqReadSimulator (https://github.com/davidliwei/RNASeqReadSimulator) to produce paired-end reads with a read length of N=151 bases, an average template length of 175 bases and a standard deviation for the template length of 25 bases, corresponding to the characteristics of the prostate cancer data we analyzed in our paper.

In order to produce different splicing efficiency levels (and hence intron retention levels) for the selected genes, we simulated 1) “full gene reads” for the entire genes (including their introns) and 2) “exon-only reads” for the exons alone (according to the GENCODE gene annotation) and then produced merged Fastq files containing both sets of simulated reads.

We defined three average target splice-junction coverage levels of 10x, 30x and 70x coverage. For each of these target coverage levels we performed 11 simulations with 0%, 10%, 20% … 100% “exon-only” reads, corresponding to average splicing efficiencies of 0, 0.1, 0.2 … 1.0, respectively. Please note, that given the high variability of RNA-seq coverage profiles, which are correctly reflected by RNASeqReadSimulator, both the coverage at splice junctions and the simulated splicing efficiencies can be considered only as average “target” values because they can vary significantly between introns.

For each simulated RNA-seq sample (i.e., for each target splice-junction coverage and target splicing efficiency), we counted what fraction of introns would be detected as (partly) retained by iREAD, IRFinder (using the threshold of $IRratio \geq 0.1$ proposed by the authors [4]) and SPLICE-q' *SE* or *IER* (using thresholds of $SE \leq 0.9$ and IER ≤ in analogy to the *IRratio* threshold because from a conceptual point of view *SE* and 1-*IRratio* can be considered as strongly related).

Fig. S5 shows that iREAD misses most of the simulated events when applied with default parameters. This is most likely due to its restrictive requirement for intron expression levels (≥3 FPKM). IRFinder and both SPLICE-q measures show mostly equivalent results but especially for lower target splice junction coverage (10x, 30x) SPLICE-q performs slightly better when identifying simulated events close to the minimum threshold (target fraction of split reads, i.e., target splicing efficiency, of 0.9).

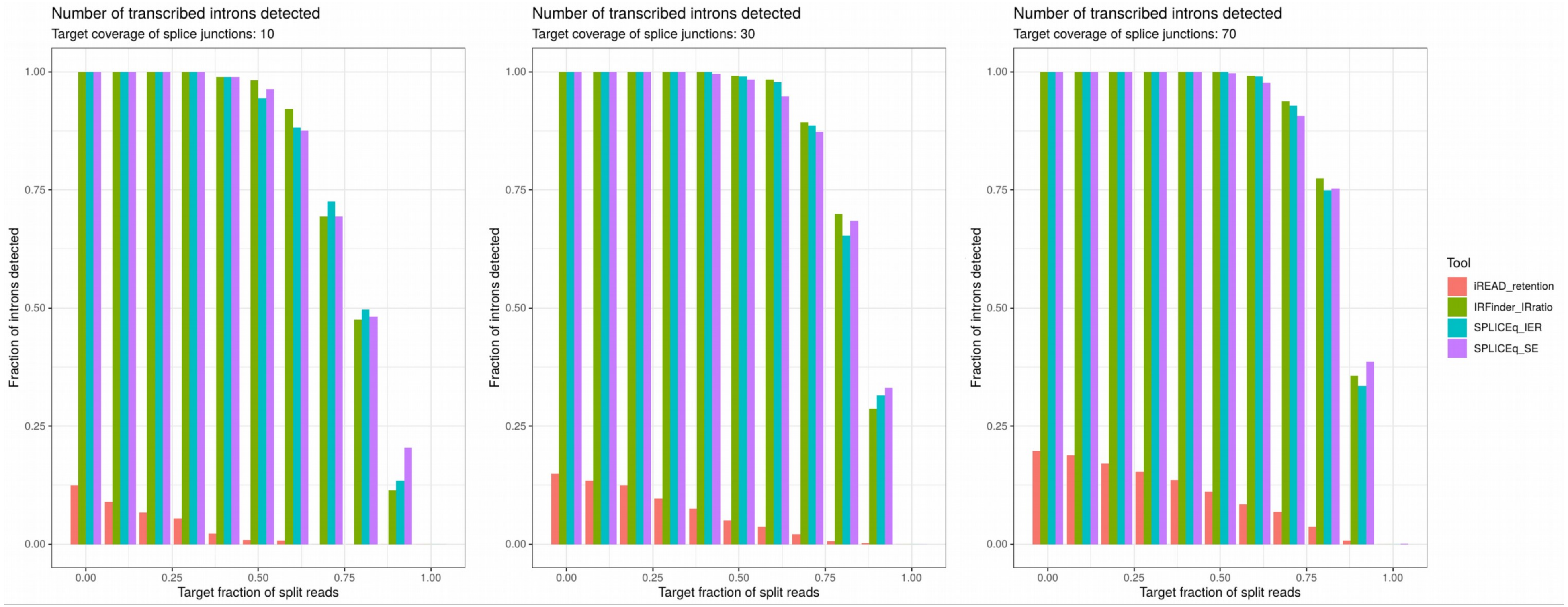

Fig. S5: Intron retention/splicing efficiency detection results with simulated RNA-seq data. The target coverage of splice junctions was 10x (left), 30x (center) or 70x (right). For each target coverage, a fraction from 0% to 100% (i.e., 0.0 to 1.0, in steps of 0.1) of the coverage were "exon-only reads", equivalent to the same average fraction of split reads at the splice junctions.

References

1. Převorovský M, Hálová M, Abrhámová K, Libus J, Folk P. Workflow for Genome-Wide Determination of Pre-mRNA Splicing Efficiency from Yeast RNA-seq Data. Biomed Res Int. 2016;2016:1–9. doi:10.1155/2016/4783841.

2. Barrass JD, Reid JEA, Huang Y, Hector RD, Sanguinetti G, Beggs JD, et al. Transcriptome-wide RNA processing kinetics revealed using extremely short 4tU labeling. Genome Biol. 2015;16:282. doi:10.1186/s13059-015-0848-1.

3. Kumar A, Badredine A, Azzag K, Kasikçi Y, Ranty MLQ, Zaidi F, et al. Patient-matched analysis identifies deregulated networks in prostate cancer to guide personalized therapeutic intervention. bioRxiv. 2019;:695999. doi:10.1101/695999.

4. Middleton R, Gao D, Thomas A, Singh B, Au A, Wong JJL, et al. IRFinder: Assessing the impact of intron retention on mammalian gene expression. Genome Biol. 2017;18:51. doi:10.1186/s13059-017-1184-4.

5. Li HD, Funk CC, Price ND. iREAD: a tool for intron retention detection from RNA-seq data. BMC Genomics. 2020 Feb 6;21(1):128. doi: 10.1186/s12864-020-6541-0.
